# A Rapid, Convergent Approach to the Identification of Exosome Inhibitors in Breast Cancer Models

**DOI:** 10.7150/ntno.73606

**Published:** 2023-01-01

**Authors:** Zoraida Andreu, Esther Masiá, David Charbonnier, María J. Vicent

**Affiliations:** Polymer Therapeutics Lab., Prince Felipe Research Center (CIPF), C. Eduardo Primo Yúfera 3, 46012, Valencia, Spain.

**Keywords:** Exosomes, ExoScreen, LBPA, Tumor microenvironment

## Abstract

Targeting cancer cell exosome release and biogenesis represents a potentially efficient means to treat tumors and prevent cancer recurrence/metastasis; however, the complexity and time-consuming nature of currently employed methods to purify and characterize exosomes represent obstacles to progression. Herein, we describe a rapid, convergent, and cost-efficient strategy to analyze candidate U.S. Food and Drug Administration (FDA)-approved drugs that inhibit exosome release and/or biogenesis using breast cancer cell line models in the hope of repurposing them for the clinical treatment of metastatic tumors. We combined the ExoScreen assay based on AlphaScreen^TM^ technology with the antibody-mediated detection of an atypical lipid (lysobisphosphatidic acid - LBPA) present in the intra-luminal vesicle/exosomal fraction to achieve both extracellular and intracellular information on exosome modulation after treatment. As proof of concept for this strategy, we identified docetaxel, biscurcumin, primaquine, and doxorubicin as potential exosome release inhibitors in the Her-2 positive MDA-MB-453 and luminal A MCF7 cell lines. Dinaciclib also functioned as an exosome release inhibitor in MCF7 cells. Further, we explored the expression of proteins involved in exosome biogenesis (TSG101, CD9 tetraspanin, Alix, SMase2) and release (Rab11, Rab27) to decipher and validate the possible molecular mechanisms of action of the identified exosome inhibitors. We anticipate that our approach could help to create robust high-throughput screening methodologies to accelerate drug repurposing when using FDA-approved compound libraries and to develop rationally-designed single/combination therapies (including nanomedicines) that can target metastasis progression by modulating exosome biogenesis or release in various tumor types.

## Introduction

**Figure 1:**
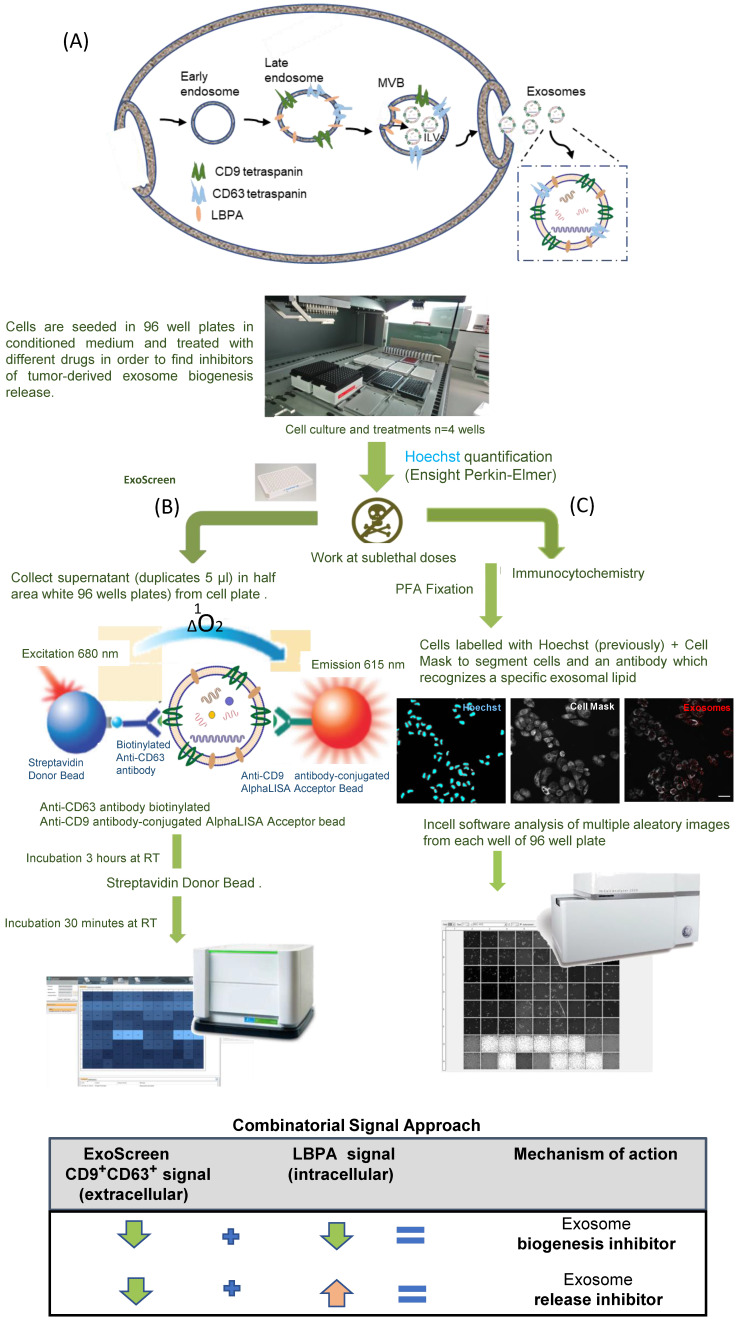
** AlphaScreen/LBPA Combination Strategy for Exosome Inhibitor Screening. (A)** Role of LBPA and tetraspanins in exosome biogenesis. Early endosomes transform into late endosomes, where LBPA and tetraspanins aid the formation of intraluminal vesicles (ILVs) by inward budding of the endosomal membrane. The resultant multivesicular body (MVB) then fuses with the plasma membrane to release ILVs into the extracellular environment as exosomes characterized by the presence of tetraspanins and LBPA.** (B)** ExoScreen assays use a tetraspanin CD9 antibody conjugated to acceptor beads and a CD63 biotinylated antibody that binds to streptavidin donor beads. A signal appears (exc. 680 nm, emi. 615 nm) if the distance between both beads is less than 200 nm (compatible with exosome size) thanks to the reactivity of O_2_. **(C)** For LBPA lipid immunocytochemistry, cells labeled with Hoechst were fixed and labeled with different markers (cell mask or phalloidin and an antibody against a specific exosomal lipid, LBPA) to allow the quantification of exosomes normalized to a single cell. (**D**) Combinatorial signal approach definition: A decrease in the ExoScreen signal together with a decrease or an increase in the LBPA InCell® fluorescence readout determines not only that the evaluated drug can inhibit exosome release but also that the reason for inhibition is an interaction with exosome biogenesis or release mechanisms, respectively.

Exosomes are extracellular vesicles (EVs) of 40-150 nm in diameter that exist within intracellular multivesicular bodies (MVBs - derived from the late endosomal compartment) as intraluminal vesicles (ILVs). ILVs become released into the extracellular space as exosomes following the fusion of MVBs with the plasma membrane (for simplicity, we employ the term “exosomes” to refer to both ILVs and exosomes unless explicitly stated) (**Figure 1A**) 1-3. Release by a range of cells leads to the presence of exosomes in many (if not all) bodily fluids, including blood plasma, serum, urine, amniotic fluid, cerebrospinal fluid, and malignant ascites 4. Exosomes play critical roles in short- and long-range cell-to-cell communication via the transfer of specific cargos (e.g., proteins, lipids, and various nucleic acid species) and pathological processes such as cancer and neurodegenerative disease 5-7. Tumor cells release large numbers of exosomes to promote the spread of disease to distant sites by promoting pre-metastatic niche formation 8: moreover, exosomes also induce immunosuppression and drug resistance to promote disease progression.

Given the crucial role of exosomes in the tumor microenvironment and metastatic progression 9, 10, the inhibition of exosome biogenesis or release pathways alone or in combination with traditional anti-tumor treatment strategies may represent a means to improve long-term healthy survival in cancer patients; however, the general lack of rapid, straightforward, and standardized techniques to isolate and characterize exosomes has frustrated the search for therapeutics that modulate exosome biogenesis or release (for simplicity, we refer to such therapeutics as “exosome inhibitors”). Conventional methods for the isolation/purification and quantification of exosomes, which include ultracentrifugation-based techniques (UC) and nanoparticle tracking analysis (NTA) 11, 12, represent time-consuming and challenging techniques to translate to high-throughput screening (HTS) and into clinical practice 11-15.

We currently lack specific markers that support exosome identification/isolation; however, studies have highlighted the enrichment of the CD9, CD63, CD81, and CD82 tetraspanin transmembrane proteins in exosomes, with CD9 and CD63 playing critical roles in exosome formation 16. While CD9 has been described as membrane-resident, CD63 mainly exists in endosomal compartments 17-19 and has been posited as a potential target for exosome isolation 20. CD63 and CD9 comprise two of the most abundant exosomal proteins, alongside, for example, the MVB biogenesis proteins Programmed Cell Death 6 Interacting Protein (PDCD6IP, also known as ALIX) and tumor susceptibility gene 101 (TSG101) (exocarta.org/exosomemarkers) 21.

The lipidic content of exosomes plays a crucial role in biogenesis and may also represent a target for exosome isolation 22. MVB internal membranes display the specific enrichment of lyso(bis)phosphatidic acid (LBPA; also known as phospholipid bis(monoacylglycero)phosphate or BMP) 23, an atypical cone-shaped phospholipid that fosters ILV formation, known as a canonical late endosome-lysosome marker 24-26. Several studies supporting the participation of LBPA in exosome formation have described the interaction of LBPA with ALIX in the control of ILV biogenesis and their participation in the sorting of tetraspanins into exosomes 24, 27, 28; moreover, a recent study described the co-localization of LBPA with Alix and CD63 and their possible use as a marker of urinary exosomes 29-33. While a small fraction of LBPA may also be found in lysosome-associated ILVs 29, we sought to take advantage of LBPA's specificity to explore the impact of exosome inhibitors on breast cancer cells. A deeper understanding of the mechanism of action of exosome inhibitors could contribute to the elaboration of additional studies and the development of novel anti-cancer therapies focusing on metastatic processes.

To facilitate the discovery of exosome inhibitors in cells and bring exosome analysis closer to clinical application, we optimized a dual-platform approach that combines the ExoScreen assay by AlphaScreen^TM^ technology (based on the research of Yoshioka *et al.* 34) with the immunocytochemical detection of LBPA; this dual approach identifies not only extracellular events but also provides crucial information regarding the intracellular cascades triggered after treatment. ExoScreen allows for the highly-sensitive analysis of protein-protein interacttions, and we employed this technology to capture and quantify exosomes released into the supernatant by cells following various treatments by detecting the presence of two exosome-enriched tetraspanins, CD9 and CD63, within a distance of 200 nm of each other (a distance compatible with the size of the exosomes) in a 96-well-plate format (**Figure 1B**) 34, 35. We then employed the immunocytochemical analysis of intracellular levels of LBPA on the same treated cells using a monoclonal antibody to label and quantify ILVs that primarily undergo release into the extracellular space as exosomes. Integrating both approaches fosters an understanding of the exosomal pathways modulated by small-molecule inhibitors (**Figure 1C**). For example, a small molecule that inhibits exosome release will lead to a low extracellular level of exosomes detected by ExoScreen and elevated intracellular LBPA levels; however, an exosome biogenesis inhibitor will lead to a low extracellular level of exosomes coupled with low intracellular LBPA levels. While many studies have employed both techniques separately to study exosome biogenesis/release 28, 30, 34-36, we now describe the combination of these techniques as a rapid and reliable means to identify exosome inhibitors and simultaneously understand their mechanism of action. We believe this convergent approach could accelerate, for example, future drug repurposing strategies when implemented with U.S. Food and Drug Administration (FDA)-approved drugs. Drug repurposing represents an attractive strategy to encounter new applications for approved drugs outside of their original scope; furthermore, this strategy offers an immediate clinical impact at a lower cost than conventional drug discovery and development. We have generally lacked a systematic effort to identify new opportunities for drug repurposing; this may be due, in part, to the lack of a comprehensive knowledge base on relevant biological targets 37. We believe our convergent approach, which can identify drugs and simultaneously elucidate possible molecular mechanisms, could help to solve this problem.

By implementing the described convergent approach, our preliminary efforts highlight the roles of conventional FDA-approved drugs (including docetaxel, doxorubicin, primaquine, or dinaciclib) as exosome inhibitors in breast cancer cell models. The small molecules evaluated in this study are widely used in clinical practice; hence, their repurposed use as antimetastatic drugs, if capable of modulating tumor-derived exosome biogenesis or release, would be of clinical interest as single agents or in combination regimes that target the primary tumor and the exosome-supported development of metastasis 38. We also analyzed isolated/purified exosomal fractions through more traditional techniques (UC and NTA) following treatment to validate our findings and delineate potential targets and mechanisms of action of candidate exosome inhibitors. We believe that this combinatorial approach will accelerate future drug discovery efforts and identify drug combinations that could represent direct treatments or form part of rationally-designed nanomedicines that target the tumor microenvironment to treat/prevent breast cancer tumorigenesis and metastasis.

## Materials and methods

### Cell Culture

**Figure 2:**
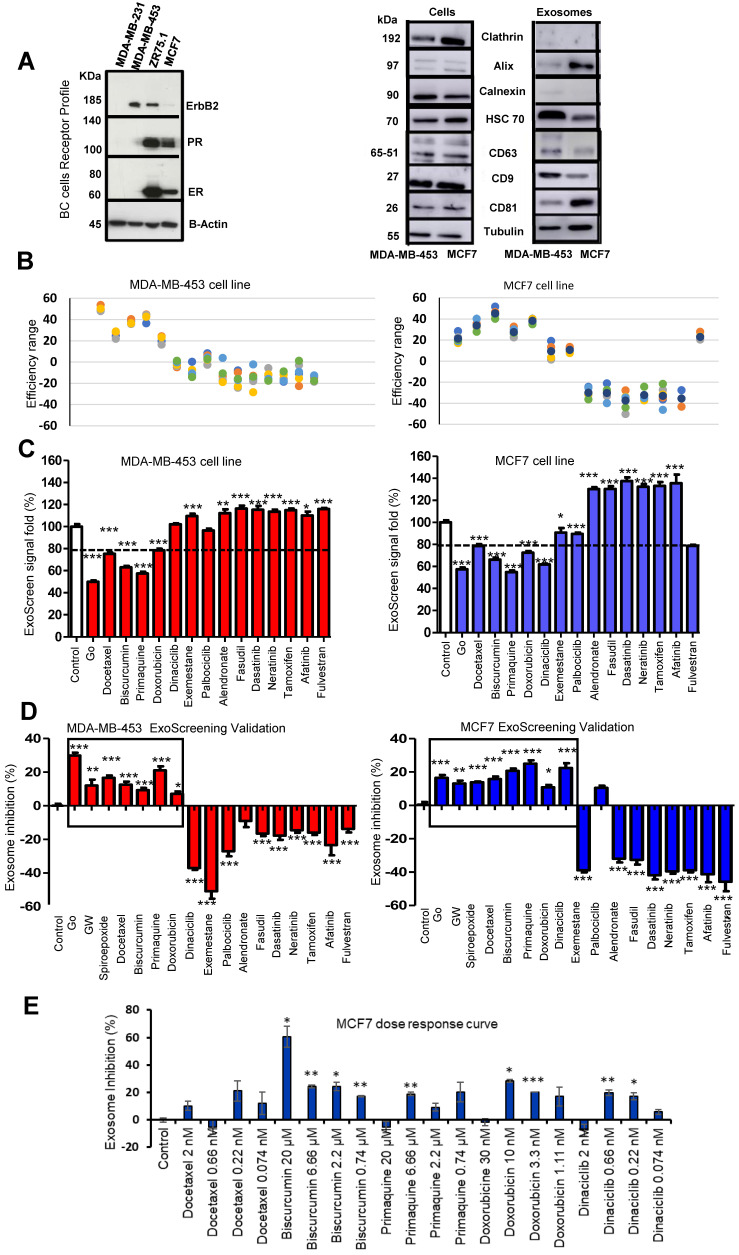
**ExoScreen-mediated Identification of Exosome Inhibitors (A)** Western blotting analysis of the receptor profile (left) of breast cancer (BC) cells and the presence of CD9 and CD63 in MDA-MB-453 and MCF7 cells and derived exosomes (isolated by UC) (right). **(B)** Plot demonstrating the efficiency range of evaluated FDA-approved small molecules; each colored dot represents a replicate (N=6 per compound tested). **(C)** ExoScreen signal detection cut-off value used to classify small molecules as exosome inhibitors/activators. Small molecules were classified as exosome inhibitors when the detected signal falls below 20% of the control signal. **(D)** ExoScreen of fourteen FDA-approved small molecules in MDA-MB-453 and MCF7 breast cancer cells (n=6 culture wells, duplicates intra-assay). Toxicity set to below 15%. Results provided four hits for MDA-MB-453 cells (docetaxel, biscurcumin, primaquine, and doxorubicin) and five hits in MCF7 cells (docetaxel, biscurcumin, primaquine, doxorubicin, and dinaciclib) as exosome inhibitors. Go6983 (Go), GW4869, and spiroepoxide used as controls for the inhibition of exosome biogenesis/release. **(E)** Analysis of the dose-dependent effect of small molecules via ExoScreen in MCF7 cells. Data depicted as mean ± SEM from Anova Dunnett's Multiple Comparison test relative to control: *p<0.05, **p<0.01, ***p<0.001, with n=3 independent experiments.

The human breast cancer cell lines MDA-MB-453 (ATCC HTB-131^TM^ - estrogen receptor (ER)-negative, progesterone receptor (PR)-negative, HER2-positive) and MCF7 (ATCC HTB-22^TM^ - ER+, PR+, HER-) were purchased from the American Type Culture Collection (ATCC, USA) and authenticated by cell genotyping at Eurofins Genomics (Europe; certificate in **Supporting Information**). Receptor expression was also profiled and confirmed by Western blotting (**Figure 2A**). Cells were routinely maintained as a monolayer in Dulbecco's Modified Eagle's Medium DMEM/F12 supplemented with 10% exosome-depleted fetal bovine serum (FBS, Hyclone, GE Healthcare Life Science, UT, USA) and 1% penicillin-streptomycin (Gibco, ThermoFisher Scientific, MA, USA) in a humidified incubator with 5% CO_2_ at 37 ºC. To deplete exosomes, FBS was diluted in DMEM/F12 (20%-80% respectively) and ultracentrifuged at 100,000 g for 17 h at 4 ºC in a Type 45 Ti fixed-angle titanium rotor (Beckman Coulter, USA). The supernatant was filtered through a 0.22 μm membrane, and the medium was further diluted with DMEM/F12 to create a 10% FBS medium with 1% penicillin-streptomycin (“exosome-free medium” prepared as described in Thery et al. 39) (**Figure S1A**).

### Application of ExoScreen: AlphaScreen^TM^ Technology to Exosomes

CD9 and CD63 detection requires two bead types: streptavidin-coated photosensitizer-containing donor beads that bind to an analyte-specific biotinylated antibody (i.e., anti-CD63) and AlphaLISA acceptor beads conjugated to a second antibody (i.e., anti-CD9) (**Figure 1A**). Upon illumination at 680 nm, donor beads detecting the CD63 epitope convert ambient oxygen into a reactive form of O_2_ that diffuses a maximum of 200 nm in solution. The emission of light at 615 nm occurs if the AlphaLISA acceptor beads lie within this short distance (if the bead-conjugated anti-CD9 acceptor bead recognizes its epitope near to the CD63 epitope); however, if the distance between acceptor and donor beads is higher than 200 nm, the singlet oxygen falls to a basal state and fails to emit light.

5 µl of cell supernatant (control and treated conditions, n=6 wells for each condition) were transferred to a 96-well white 1/2 area microplate (Perkin Elmer, Madrid, Spain). Samples were incubated for 60 min at room temperature (RT) with 10 µl/well anti-human CD9 antibody (SHI-EXO-M01-50; CosmoBio Co, Tokyo, Japan) conjugated to AlphaLisa acceptor beads (10 µg/ml; 6772001, Perkin Elmer, Madrid, Spain) and 10 µl/well biotinylated human anti-CD63 antibody (0.3 nM, SHI-EXO-M02-50, CosmoBio Co, Tokyo, Japan). 25 µl/well of AlphaScreen^TM^ streptavidin-coated donor beads (40 µg/ml; 6760002, Perkin Elmer, Madrid, Spain) were then added and incubated for another 30 min at RT in the absence of light. Any signal was detected using an EnSight multimode plate reader (Perkin Elmer, Madrid, Spain) (**Figure 1B**). The results derived from each well were normalized to cell viability (nuclei numbers), as previously determined via cell labeling with Hoechst 33342 (2 µg/ml) (ThermoFisher Scientific, MA USA). The nuclei images were obtained with the same microplate reader, using a 4x magnification objective.

The conjugation of the human CD9 antibody to beads was performed as follows: beads (25 µl at 20 mg/mL) were washed with 50 µl of PBS in an Eppendorf and then centrifuged at 16,000 g for 15 min. The supernatant was then removed, and the CD9 antibody was added to the beads (0.05 µg antibody per 0.5 µg beads), followed by the addition of sodium phosphate (130 mM, pH 8), 10% Tween 20, and sodium cyanoborohydride (400 mM). The beads were resuspended by pipetting and incubated at 37ºC while rotating at 300 rpm for 48 h to improve performance. To improve stability, a blocking step was performed; 10 µl of a fresh solution of carboxymethoxylamine (65 mg/mL) in 800 mM NaOH was added to the conjugation reaction to block unreacted sites and incubated on a rotor for 60 min at 37 ºC and 300 rpm. The conjugation reaction was then centrifuged for 15 min at 16,000g at 4 ºC, the supernatant removed, and the pellet resuspended in 200 µl of Tris-HCl (100 mM, pH 8) and washed twice in PBS. Finally, the pellet was resuspended in 0.05% Proclin300 in PBS as a preservative agent, vortexed, sonicated for 10 seconds, and preserved at 4 ºC in the absence of light. To prepare the biotinylated human CD63 antibody stock, 45 µg of CD63 antibody was resuspended in 96.2 µl of PBS and 3.8 µl of BiotinChromaLink (2 mg/ml) solubilized in anhydrous dimethylformamide (DMF). **Figure S1 (B-G)** provides additional information regarding the optimization of the ExoScreen assay.

### LBPA Immunostaining Using InCell Analyzer

Cells were seeded in black, clear-bottomed 96-well plates for LBPA immunostaining. After removing the supernatant (for the ExoScreen assay), cells were incubated with Hoechst 33342 (2 µg/mL) (ThermoFisher Scientific, USA) to label nuclei and fixed in 1% paraformaldehyde (Electron Microscopy Sciences, USA) for 10 min at RT (100 µl/well). Cells were then permeabilized with saponin 5% w/v (Alfa Aesar A18820; ThermoFisher, USA) to facilitate the penetration of the primary antibody and blocked with PBS and 1% bovine serum albumin (BSA; A7906, Sigma-Aldrich, Spain) to prevent non-specific binding. Cells were then incubated with a primary antibody against LBPA (purified mouse monoclonal anti-LBPA [BMP], 1:100, Echelon, USA) overnight at 4ºC and then with an appropriate secondary antibody (goat anti-mouse IgG H&L Alexa Fluor® 488 [ab150113], 1:500, Abcam, UK) for 1 h at RT in the absence of light. Finally, cells were labeled with phalloidin (phalloidin-tetramethylrhodamine B isothiocyanate, 50 µg/ml, Sigma-Aldrich, Spain) for 10 min at RT in the absence of light to allow nucleus/cytoplasm segmentation and assure the association of LBPA exosome signal to a single cell in subsequent analysis. Cells were washed twice with PBS for 10 min between each immunocytochemistry step (**Figure 1C** and **Figure S2**).

Data were acquired using an InCell® Analyzer 2200 instrument (GE Healthcare, UK) comprising an inverted epifluorescence microscope equipped with a solid-state illumination source and different objective and excitation/emission filters. The images were collected using a 16-bit sCMOS camera. Image acquisition employed three pairs of excitation/emission dichroic filters: 390/18 excitation and 432.5/48 emission for Hoechst, 475/28 excitation and 511.5/23 emission for LBPA-FITC (fluorescein isothiocyanate), and 542/27 excitation and 597/45 emission for Phalloidin-TRITC (tetramethylrhodamine). A 20x/0.45 NA objective was used to collect twenty images per well. After data acquisition, images were analyzed in the Developer Toolbox software (GE Healthcare, UK). The analysis workflow involved nuclei and cell segmentation based on Hoechst and phalloidin staining, respectively. LBPA granules were segmented using the FITC signal and linked with cell segmentation to quantify the granules inside the cells and avoid analyzing artifacts. **Figure S2** shows representative images of the workflow segmentation in the LBPA assay. Segmentation steps and mathematical algorithms were applied to quantify the number of granules per cell, the number of cells with granules, the area, and the intensity of granules per cell. Finally, exosomal number (LBPA signal) was normalized to the number of nuclei to obtain the number of granules per cell. The LBPA signal registered by equipment represents the FITC intensity of granules by cell. Results are shown as the percentage of LBPA-positive granules per cell. The number of LBPA-positive granules per cell detected by the InCell® Analyzer 2200 in the control condition (cells without treatment) was established as a reference and considered 100% LBPA-positive granules per cell. LBPA signals for treatments were normalized to the control condition.

### Convergent Screening Approach towards the Identification and Characterization of Small Molecules as Tumor-associated Exosome Modulators

All small molecules employed were purchased from Sigma-Aldrich (USA) and MedKoo Biosciences (China). Cells were seeded at a density of 31,250 cells per cm^2^ (MDA-MB-453) and 15,625 cells per cm^2^ (MCF7) in black with clear bottom 96-well plates in exosome-free media and allowed to settle overnight. Selected FDA-approved drugs were added at non-toxic concentrations (less than 20% toxicity) and incubated for 72 h. The working concentrations for each small molecule were previously determined by CellTiter 96® Aqueous Non-Radioactive Cell Proliferation Assay, carried out according to the manufacturer's protocol (Promega, Wisconsin, USA) (**Figure S3).** At 72 h post-treatment, the cell supernatant was removed and used for ExoScreen assays; meanwhile, the remaining cells were fixed and used for LBPA signal detection by immunocytochemistry. All small molecules were solubilized in dimethyl sulfoxide (DMSO) and later compared with their specific vehicle as control (except for alendronate, which was solubilized in PBS, thereby representing the control in this case). **Tables S1** and **S2** show detailed information on the experimental conditions and results obtained from our combinatorial signal. Measurements were performed in triplicate, and the Z´-factor determined the assay quality and robustness. The Z'-factor is defined in terms of four parameters: the mean (μ) and standard deviation (σ) of both the positive (p) and negative (n) controls (μp, σp μn, σn) 40. Given these values, the Z'-factor is defined as:



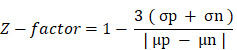



### Validation Assays for Exosome Characterization and Quantification Post-treatment via Traditional Approaches

#### (a) Exosome Isolation by Ultracentrifugation

To purify exosomes from cell-conditioned media, breast cancer cells were first cultured in media supplemented with 10% exosome-depleted FBS in 150 cm^2^ plates (P150). Cells were allowed to settle overnight and treated for 72 h with the candidate exosome inhibitors identified by ExoScreen. Supernatant fractions were collected from three P150 plates after 96 h of cell culture for each condition (control and treatments) and pelleted by centrifugation at 400 g for 5 min. The supernatant was transferred to Ultra-Clear centrifuge tubes (Beckman Coulter, Madrid, Spain) and centrifuged at 20,000 g for 30 min at 4 ºC in a Hitachi ultracentrifuge-CP100NX (Hitachi Power Tools Iberica, S.A., Barcelona, Spain) using a Type 45 Ti fixed-angle titanium rotor (Beckman Coulter, Madrid Spain). The exosome supernatant was then transferred to new centrifuge tubes and centrifuged at 100,000 g for 70 min. The exosome pellet fraction was washed with 20 mL of PBS and centrifuged again under the same conditions but in smaller ultra-clear centrifuge tubes and a 50.2 Ti type rotor (Beckman Coulter, Madrid, Spain). Finally, the pellet was resuspended in 100 µl of PBS for subsequent characterization and quantification (**Figure S4**).

#### (b) Exosome Characterization by Nanoparticle Tracking Analysis

Nanoparticle tracking analysis (NTA) employed a NanoSight NS300 (Malvern Panalytical, Malvern, UK) equipped with fluorescence detection (488 nm filter) and an automatic syringe pump system. Five 30-second videos were recorded for each sample with a camera level set at 10 and a detection threshold set at 5. The temperature was maintained at 25ºC throughout the measurement process. Videos recorded for each sample were analyzed with NTA software (Version 3.1) to determine the concentration and size of measured particles with the corresponding standard error (**Figure S4C**). Before injecting into the NanoSight, samples were diluted (1:100-1:200) to achieve a concentration of work between 10^8^-10^9^ particles/ml. Validated patterned silica microspheres (0.1-0.5 µm, Polysciences Europe GMBH) were used to calibrate the equipment before use to ensure NTA accuracy.

#### (c) Exosome Characterization by Transmission Electron Microscopy

For transmission electron microscopy (TEM) studies, exosome samples obtained by UC from an equal amount of culture media were adsorbed on carbon-coated nickel grids by floating an ionized grid onto a drop of the sample. The grids were contrasted with 2% uranyl acetate. In the case of cell samples, cells were seeded in Lab-Tek chamber slides from two wells (Nalge Nunc International, IL, USA) and fixed in 3% glutaraldehyde in 0.1 M phosphate buffer (PB) for 2 h at 37 ºC. Lastly, samples were washed five times in 0.1 M PB and stored at 4ºC. The samples were post-fixed in 2% OsO_4_ for 60 min at RT and stained in 2% uranyl acetate in the absence of light for 2 h at 4 ºC. Samples were then rinsed in distilled water, dehydrated in ethanol, and embedded overnight in Durcupan resin (Sigma-Aldrich, USA). Following polymerization, embedded cultures were detached from the wells and glued to Durcupan blocks. Finally, ultrathin sections (0.08 µm) were cut with an Ultracut UC-6 (Leica microsystems, Germany) and stained with lead citrate (Reynold's solution). To enumerate exosomes, seven fields were randomly counted from each grill (n=3) by tracing a right-left path and starting again one field lower so as not to repeat counted fields. Vesicles with a cup shape (morphology described for exosomes) were counted. The data obtained in each field were added together, and the result expressed as the number of exosomes per field. Exosomal and cell samples were examined with an FEI Tecnai Spirit BioTwin transmission electron microscope (ThermoFisher Scientific, USA) using a Morada digital camera (EMSIS GmbH, Germany).

### Elucidating the Molecular Mechanisms of Action of the Identified Exosome Inhibitors by SDS-PAGE and Western Blotting

Equal amounts of cell or exosomal lysates were resuspended in 4X loading buffer and then denatured at 95 ºC for 7 min. Protein extracts were resolved by SDS-PAGE in a 10% polyacrylamide gel (Acryl/Bis^TM^ 29:1 UltraPure VWR Life Science, Ohio USA) and transferred onto a polyvinylidene difluoride (PVDF) membrane (Thermo Scientific, USA). Membranes were incubated with anti-ERα (D8H8) rabbit monoclonal antibody (8644), anti-PR (C1A2) (both from Cell Signaling, Leiden, The Netherlands), anti-ErbB2 (HER2) rabbit polyclonal antibody (BioVision, USA), and anti-β-actin mouse monoclonal antibody clone AC-15 (Sigma-Aldrich, Spain) for the verification of breast cancer cell status (all at a dilution of 1:5000). Membranes were incubated with anti-CD9 and anti-CD63 (ExoAB antibody kit (EXOAB-KIT-1), System Biosciences, CA, USA; dil. 1:4000) for cellular and exosomal analysis, Rab27A polyclonal antibody (LabClinics, 66058-1-Ig; dil. 1:4000), Rab11A polyclonal antibody (Abcam, ab652000; dil. 1:4000), N-SMase2 ((G-6): sc-166637, Santa Cruz Biotechnology, INC; dil. 1:2500), Alix ((3A9), mouse monoclonal antibody, cell signal; dil. 1:2000); TSG 101 ((C-2): sc-7964 Santa Cruz Biotechnology, INC; dil. 1:2500), and anti-mouse or rabbit secondary antibodies as appropriate at dil. 1:20000 (Sigma-Aldrich). This step was followed by the addition of peroxidase (HRP) coupled with secondary antibodies and detection by chemiluminescence with Amersham Hyperfilm^TM^ MP (GE Healthcare Limited, UK) using a CURIX 60 machine (AGFA, Electromedinter S.L., Madrid, Spain).

### Statistical Analysis

Data from three independent assays are represented as the mean ± SEM or mean ± SD (specified in each case). In the case of ExoScreen and InCell® analysis, six or more wells were analyzed to provide reliable results. A representative blot is provided for all Western-based protein analyses. Statistical analyses were performed using GraphPad Prism5 (GraphPad Software Inc., La Jolla, CA, USA). Analysis of variance (ANOVA) was performed using Dunnett's Multiple Comparison test relative to the control condition. Differences were considered statistically significant if the P-value was equal to or less than 0.05 (*p<0.05, ** p<0.01, *** p<0.001).

## Results

Identifying molecules that can modulate tumor-associated exosome release and/or biogenesis represents a potential means to prevent/treat cancer and associated metastasis 2, 10. Towards this aim, we report a convergent combinatorial approach for drug identification (**Figure 1**) based on already reported ExoScreen technology 34 with the ability to simultaneously elucidate the molecule mechanism of action ruling exome modulation in each case. For this purpose, we combined two techniques - one based on Alpha (Amplified Luminescence Proximity Homogeneous Assay) technology 34 and the other on the identification of a specific exosomal lipid by immunocytochemistry 30. As a first step toward developing the screening method, we aimed to demonstrate validity and robustness; therefore, we selected fourteen well-known FDA-approved drugs (**Tables S1** and **S2**) that interfere with molecular mechanisms involved in exosome modulation. This list includes drugs that interfere with microtubule stability, reduce exosome immune suppressive activities against natural killer cells, modulate the pH of the tumor microenvironment, inhibit the Rho/Rock pathway, or interfere with the sorting (sphingomyelinase inhibitors) and expression of endosomal proteins (including Rabs) 2, 3, 9, 41.

### ExoScreen-mediated Identification of Exosome Inhibitors

We selected the Her-2 positive MDA-MB-453 and luminal A MCF7 cell lines as representative examples of hormone-independent and -dependent breast cancer cells. We first confirmed the presence of CD9 and CD63 in cells and exosomes by Western blotting (**Figure 2A**). Importantly, the expression of common exosomal proteins (e.g., Alix, HSC70, and CD81) and the absence of cell proteins (e.g., the plasma membrane protein clathrin or the endoplasmic reticulum protein calnexin) indicate that the isolated exosome population does not suffer from the presence of contaminating vesicles from other cell compartments **(Figure 2A)**. Additionally, we optimized the parameters used in our ExoScreen, including the cell media (**Figure S1A**) and adequate signal intensity (**Figure S1B-C**). The absence of differences in ExoScreen signal before and after centrifugation demonstrated that cell debris or other larger vesicles did not interfere with the ExoScreen results, thereby suggesting assay suitability (**Figure S1D**). Using cell viability assays (**Figure S3**), we selected non-toxic working concentrations for each selected FDA-approved small molecule to ensure that a decrease in the ExoScreen signal during the experiment solely derives from decreased exosome biogenesis or release. **Figure 2B** displays the percentage change in exosome modulation activity of each small molecule evaluated. To distinguish low/high ExoScreen signals and classify small molecules as exosome inhibitors, we established an experimental cut-off value (**Figure 2C**) - we considered an ExoScreen signal below 80% as a reduced signal (inhibitors) and significantly above 120% as an increased signal (activators) when compared to control (100% signal). The protein kinase C (PKC) inhibitor Go6983 (Go) acted as a positive control 42, as PKC activation triggers an increase in intracellular calcium flux that triggers EV release. We also used neutral sphingomyelinases (N-SMases) inhibitors (GW4869 and spiroepoxide) that interfere with exosome biogenesis 43 as additional controls.

The ExoScreen assay identified four FDA-approved drugs in MDA-MB-453 cells (docetaxel, biscurcumin, primaquine, and doxorubicin) and five (docetaxel, biscurcumin, primaquine, doxorubicin, and dinaciclib) in MCF7 cells as potential exosome inhibitors (**Figure 2D**). Meanwhile, the increased ExoScreen signal detected in the cell supernatants for the remaining small molecules (exemestane, alendronate, fasudil, dasatinib, neratinib, tamoxifen, afatinib, and fulvestrant in both cell types, and dinaciclib additionally in MDA-MB-453 cells) compared to control indicated these small molecules as exosome biogenesis/release “activators”. We present data as a percentage of exosomal particles in cell supernatants after normalization to cell nuclei (represented in **Figure 2D** as the percentage inhibition for each screened small molecule). The Z' factor is commonly used in HTS (also known as Z-prime) to judge whether the pharmacological response in a particular assay is dependable enough to warrant further attention 40. The Z' factor for the different ExoScreen assays performed was always above 0.75 (calculated following the equation shown in the experimental section), indicating the optimal function of the assay and the high confidence of the results for quantitative screening of exosome inhibitors. We also analyzed dose-response in MCF7 cells to evaluate the range of concentrations in which our identified hits exert their inhibitory activity (**Figure 2E**). Overall, biscurcumin, doxorubicin, and dinaciclib displayed dose-dependent inhibition (biscurcumin at all concentrations, although doxorubicin and dinaciclib did not display this trend at the highest concentration, probably due to interference with cell death mechanisms). Docetaxel and primaquine failed to show dose dependence. In agreement with data reported for colon cancer 34, our results suggest that ExoScreen can quantify exosomes in breast cancer cell models without the requirement for laborious purification steps and can effectively identify exosome inhibitors.

### Differentiating Exosome Biogenesis from Release Inhibition via Intracellular LBPA Analysis

**Figure 3:**
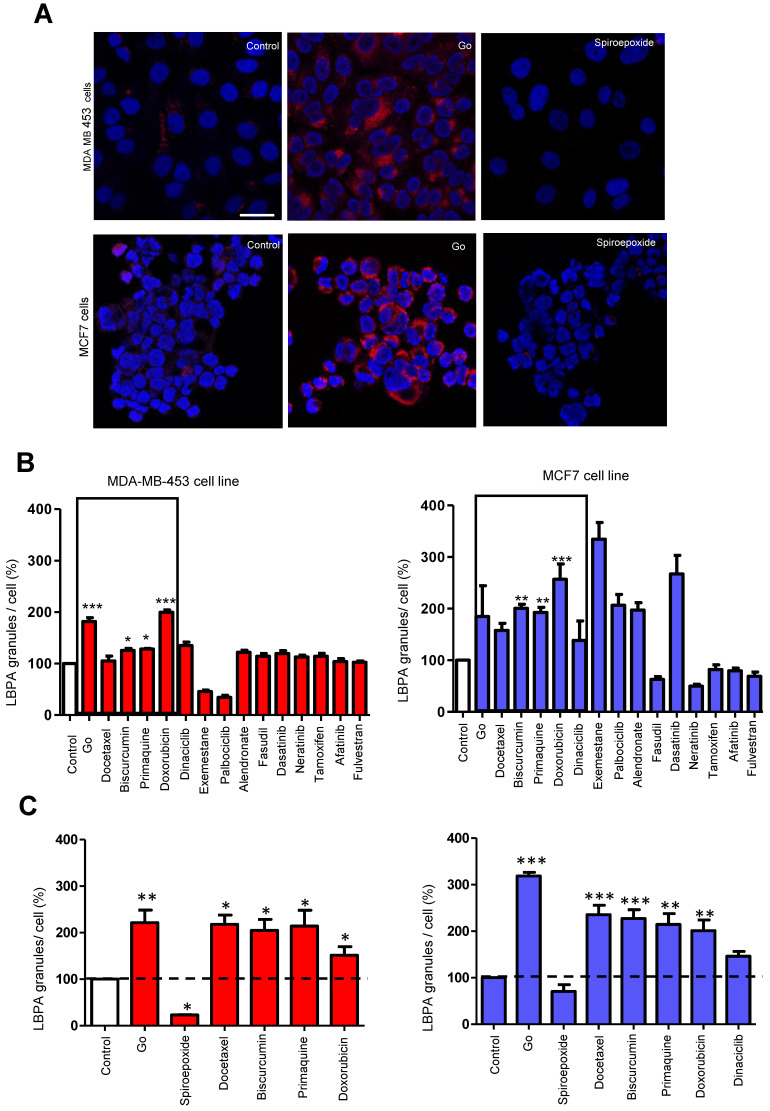

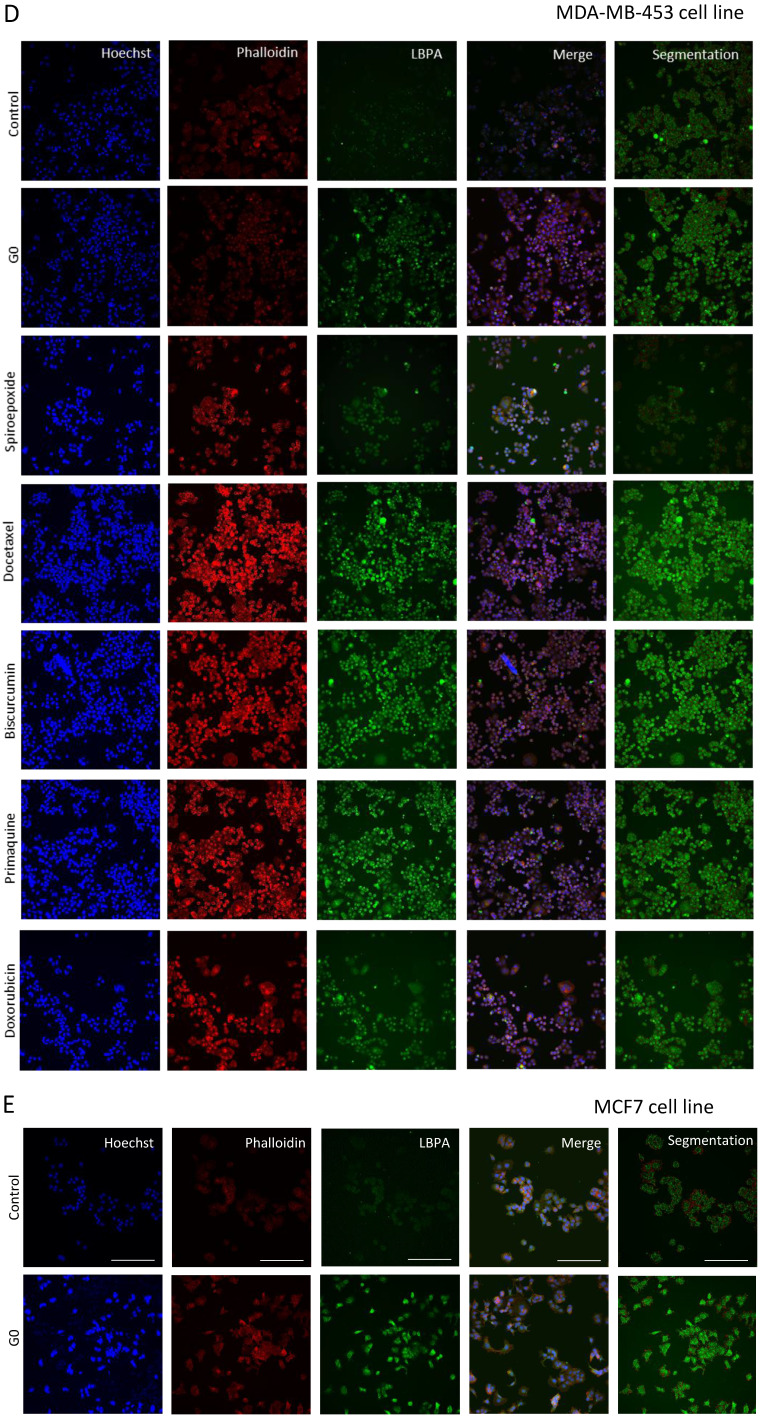

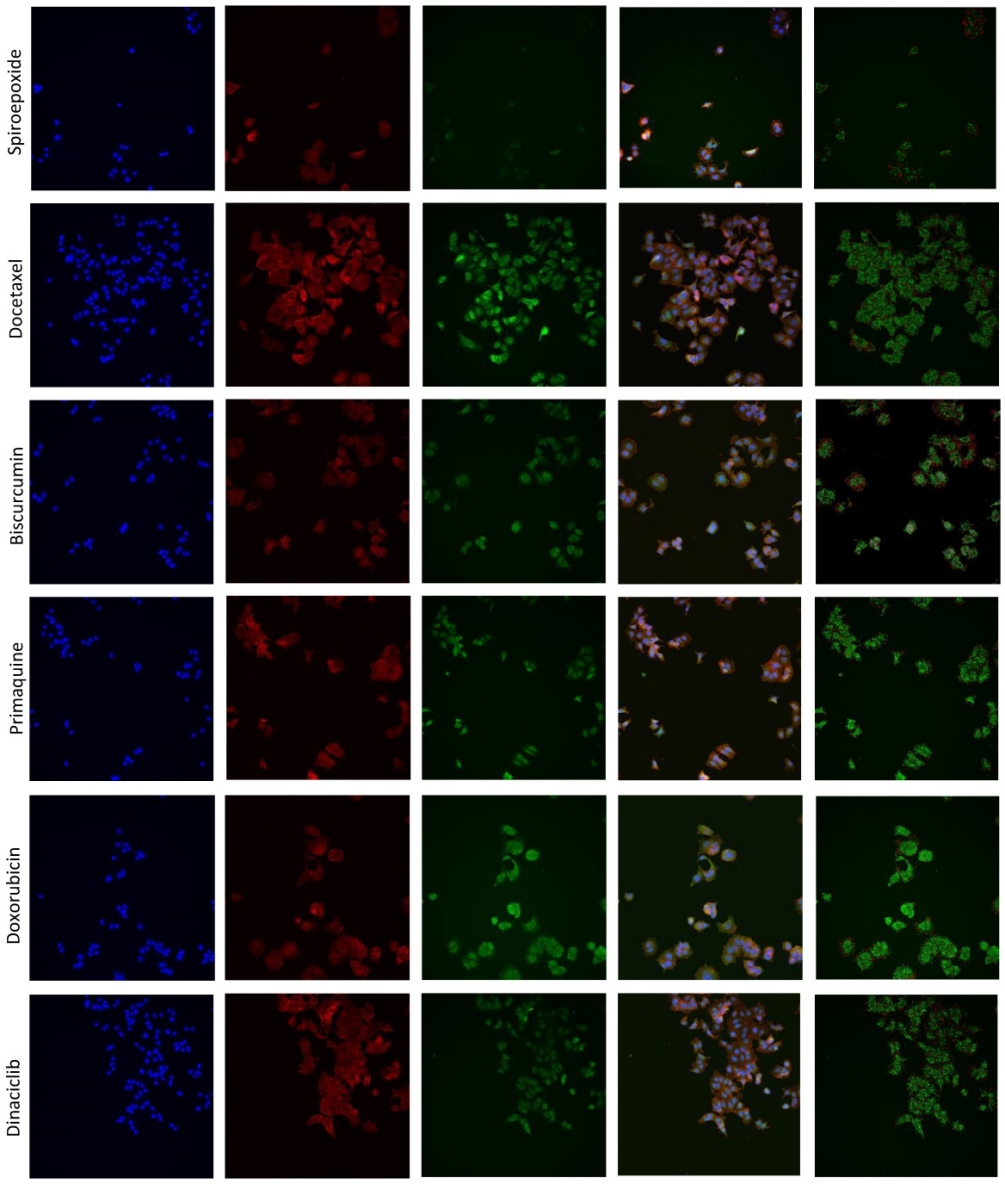
** Optimization of Exosomal LBPA Signal Detection to Decipher the Mechanism of Action of Small Molecules.** Previously described inhibitors of exosome biogenesis/release (Go6983 and spiroepoxide) were used to standardize the LBPA assay and immunostaining. **(A)** Confocal images showing exosomal LBPA signal (granules in red). A high intracellular LBPA signal following Go6983 treatment confirms the inhibition of exosome release (but not biogenesis) and the intracellular retention of exosomes. The low intracellular LBPA signal following spiroepoxide treatment confirms the inhibition of exosome biogenesis. Scale bar: 20 µm. **(B)** Quantitative analysis demonstrating LBPA results for the small molecules evaluated in the ExoScreen assay. Data shown as intracellular LBPA signals from six independent wells. An elevated number of intracellular exosomes in MDA-MB 453 cells occurs following docetaxel, biscurcumin, primaquine, and doxorubicin treatment. These small molecules and dinaciclib also promote the retention of exosomes within MCF7 cells. **(C)** Validation of intracellular LBPA signal for small molecules exosome inhibitors. **(D, E)** Representative InCell® images from intracellular LBPA signal in MDA-MB-453 and MCF7 cells, respectively. Nucleus stained in blue (Hoechst), cell stained in red (phalloidin), and exosomes stained in green (LBPA). The images in the final column on the right (red and green) show cells and exosomes segmented for analysis with phalloidin and LBPA, respectively. Go6983 and spiroepoxide used as positive controls for exosome inhibition. Data normalized to the corresponding control. Twenty random fields were photographed for each well and condition. ANOVA, Dunnett´s Multiple Comparison test relative to control condition: *p<0.05, ** p<0.01, *** p<0.001.

LBPA detection using InCell technology® 44, 45 can resolve ExoScreen analysis by discriminating the modulation of exosome release from biogenesis, thereby helping to explore underlying mechanisms of action. We optimized the LBPA immunofluorescence assay using Go6983 and spiroepoxide. Although GW4869 represents a well-known inhibitor of exosome generation, the autofluorescence of this small molecule has the potential to mask the LBPA immune signal in this assay. Treatment of breast cancer cells with Go6983 resulted in a higher intracellular LBPA signal suggesting the inhibition of release; meanwhile, spiroepoxide treatment resulted in a lower LBPA signal in agreement with its function as an exosome biogenesis inhibitor (**Figure 3A**). Transferring these experimental conditions to the InCell® Analyzer 2200 automated microscope allowed the performance of parallel assays (a step toward a desired HTS approach in the future). The image workflow analysis in the InCell® Analyzer involves nuclei and cell segmentation based on Hoechst and phalloidin staining, respectively, that quantifies the number of exosomes normalized to a single cell **(Figure S2)**. The images within the two last panel columns in **Figures 3D** and** E** depict the merge of the different cell stains and segmentation to ensure correct quantitative analyses.

The InCell® Analyzer output provided evidence for an increase in intracellular LBPA signal following docetaxel, biscurcumin, primaquine, doxorubicin, and dinaciclib treatment compared to untreated MCF7 cells. We also observed a similar profile in MDA-MB-453 cells except for dinaciclib (**Figure 3B,** validation assay** Figure 3C**). Combined with a low ExoScreen signal (**Figure 2E**), our results suggest that the above-identified FDA-approved drugs represent exosome release inhibitors. We discovered that treatment with alendronate, dasatinib, fasudil, and tamoxifen in MDA-MB-453 cells and exemestane, alendronate, or dasatinib in MCF7 cells led to a high ExoScreen signal in the cell supernatant and a high intracellular LBPA signal, suggesting these small molecules function as exosome biogenesis activators (**Figures 2C** and** 3B, Table S1** and** S2)**. Of note, the intracellular LBPA signal does not directly correlate with the ExoScreen assay results; instead, these two measurements complement each other.

**Figure 4:**
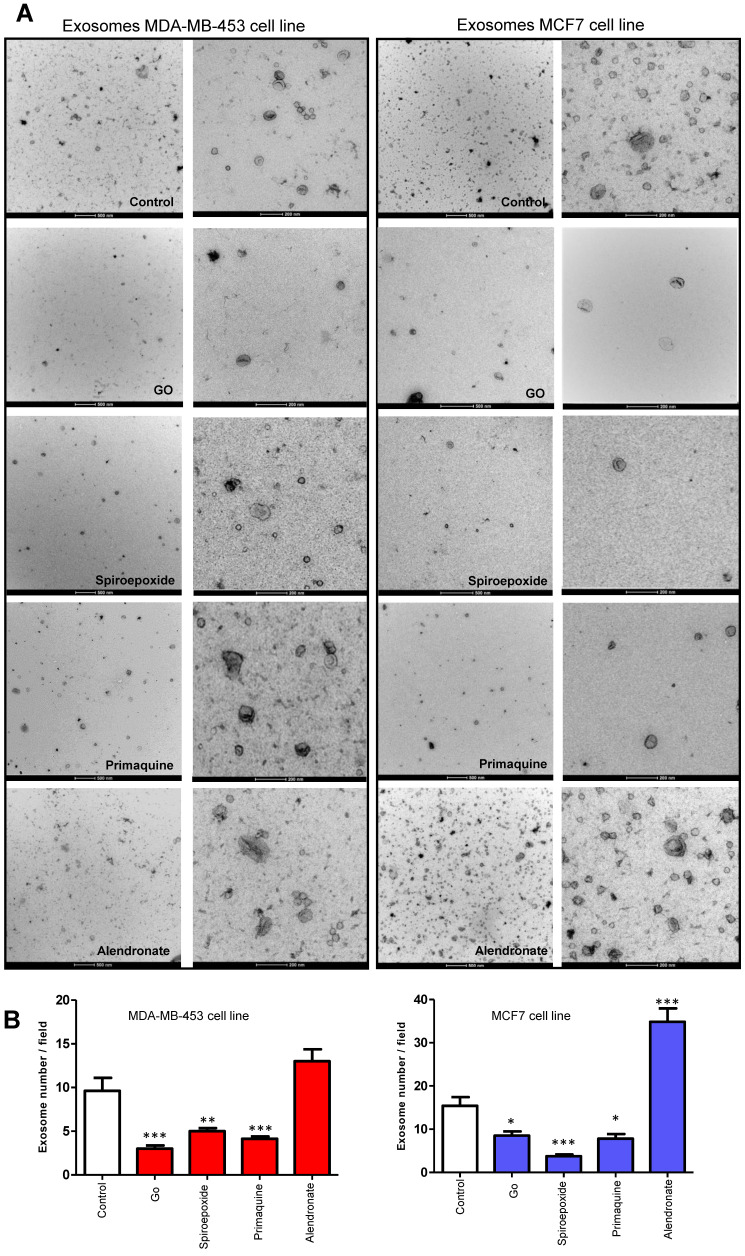

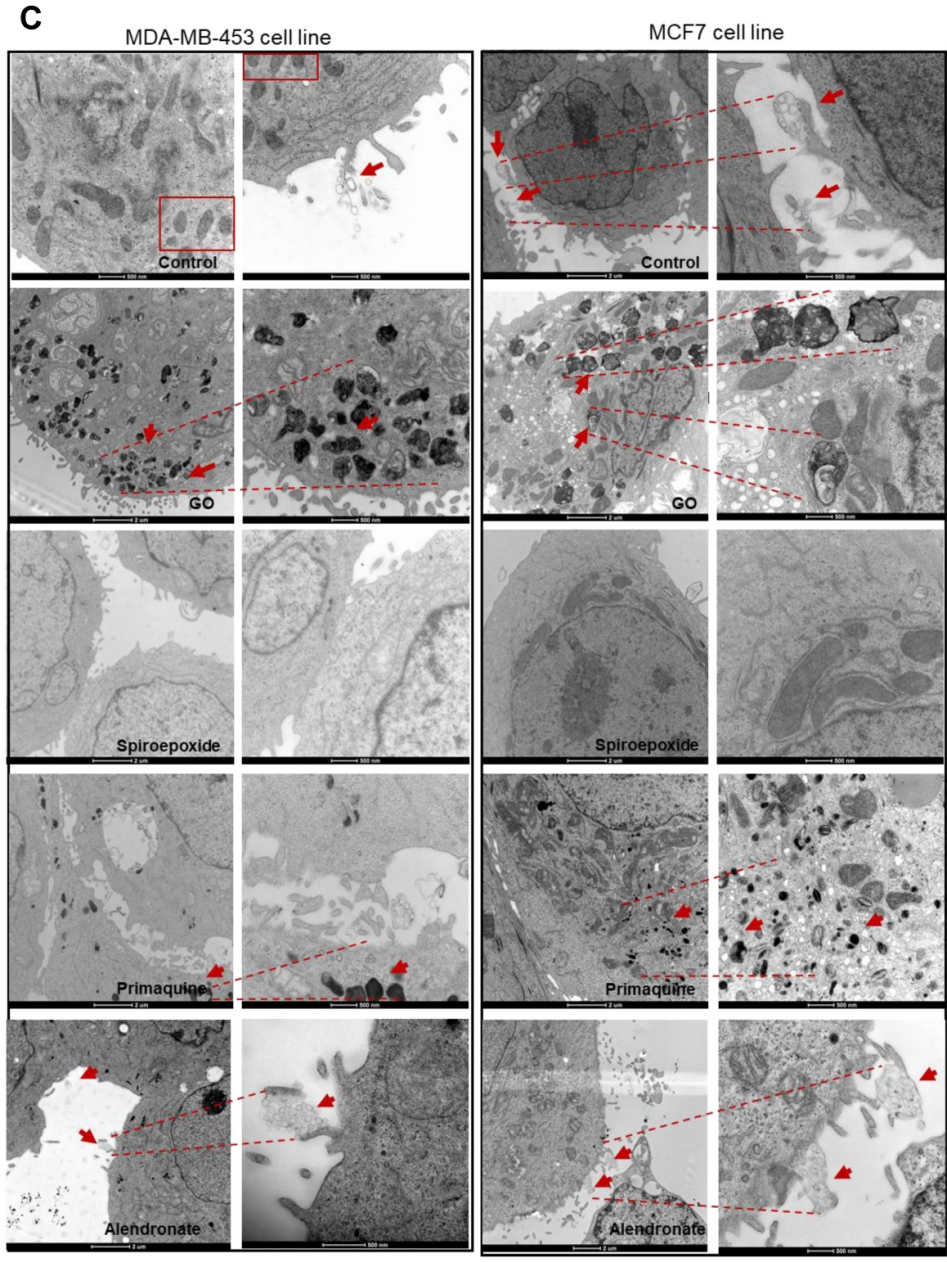
**Validating Alterations to Exosome Biogenesis/Release after Treatment. (A)** Representative TEM images of exosomes derived from MDA-MB-453 and MCF7 cells after treatment with Go6983 (Go) as an exosome release inhibitor or spiroepoxide as an exosome biogenesis inhibitor. Primaquine and alendronate are examples of exosome biogenesis/release inhibitors and activators, respectively. The presence of exosomes in the supernatant of cells treated with Go6983, spiroepoxide, and primaquine decreases, while alendronate treatment increases exosome levels. Scale bars: 500 nm and 200 nm (left and right, respectively, in each panel). (**B**) Quantitative analysis of seven different fields from TEM images. **(C)** Representative electron microscopy images in cells treated with Go6983, spiroepoxide, primaquine, and alendronate**.** Exosomes accumulated inside MDA-MB-453 and MCF7 cells after treatment with Go6983 and primaquine (exosome release inhibitors). Exosome accumulation was not observed in cells treated with spiroepoxide (exosome biogenesis inhibitor) or alendronate (activates exosome biogenesis/release). Arrows mark the presence of exosomes accumulating in MVBs and the extracellular space. Dotted lines represent the magnification of the area of interest. Scale bars: 2 µm and 500 nm (left and right, respectively, in each panel). Data depicted as mean ± SEM from Anova Dunnett's Multiple Comparison test relative to control: *p<0.05, **p<0.01, ***p<0.001.

To further support our findings, we acquired TEM images of cells treated with Go6983 and spiroepoxide and representative small molecule inhibitors and activators of exosome biogenesis/release (**Figure 4**). We observed a decrease in exosome levels after treating breast cancer cells with spiroepoxide and Go6983 as controls for the inhibition of biogenesis and release, respectively. We captured low magnification TEM images to indicate the overall differences in exosome number in each sample to underline the effectiveness of the identified hits (**Figure 4A,** left of each panel).

Quantification of data from these images demonstrated the significant differences between alendronate - an exosome release activator - and primaquine - an exosome release inhibitor compared to control untreated cells (**Figure 4B**). Image magnification (**Figure 4C**) provided evidence for intracellular exosome retention in breast cancer cells treated with release inhibitors (Go6983 and primaquine); meanwhile, we failed to observe any intracellular accumulation following treatment with the exosome biogenesis inhibitor spiroepoxide and the release activator alendronate. TEM images confirmed the reliability of our dual approach (**Figure 4C**).

### Comparison with Conventional Techniques: Confirmation of Identified Inhibitors detected by Ultracentrifugation and Nanoparticle Tracking Analysis

UC and NTA represent currently accepted techniques for exosome isolation and quantification, respectively, and we employed these techniques to validate candidate inhibitors identified by our combined ExoScreen/LBPA approach (**Figure S4**). We corroborated the ability of docetaxel, primaquine, and Go6983 in MDA-MB-453 cells and biscurcumin, primaquine, doxorubicin, and Go6983 in MCF7 cells to inhibit exosome release (**Figure S4A** and** C**); however, we failed to confirm the activity of biscurcumin and doxorubicin in MDA-MB-453 cells and docetaxel and dinaciclib in MCF7 cells by UC and NTA. These results may derive from the more sensitive nature of the ExoScreen and LBPA techniques as they assess a more defined exosome population (expressing CD9, CD63, and LBPA) and not a pool of different EVs, as occurs when employing UC. Of note, isolation methods influence the nature of the EVs evaluated 46 - exosomes isolated by UC represent a more heterogeneous population with likely co-isolation of mixed EV populations 39 compared to the population evaluated with our described approach.

Overall, we suggest that the implementation of ExoScreen technology supports the isolation of a population of EVs highly enriched in exosomes, enhancing the sensitivity of any assay and making it more useful for routine laboratory and clinical use. Further, our described technique can evaluate a higher number of samples and potentially be applied in HTS approaches following further development.

### Understanding the Molecular Mechanisms of Action Impacted by Exosome Inhibitors

**Figure 5:**
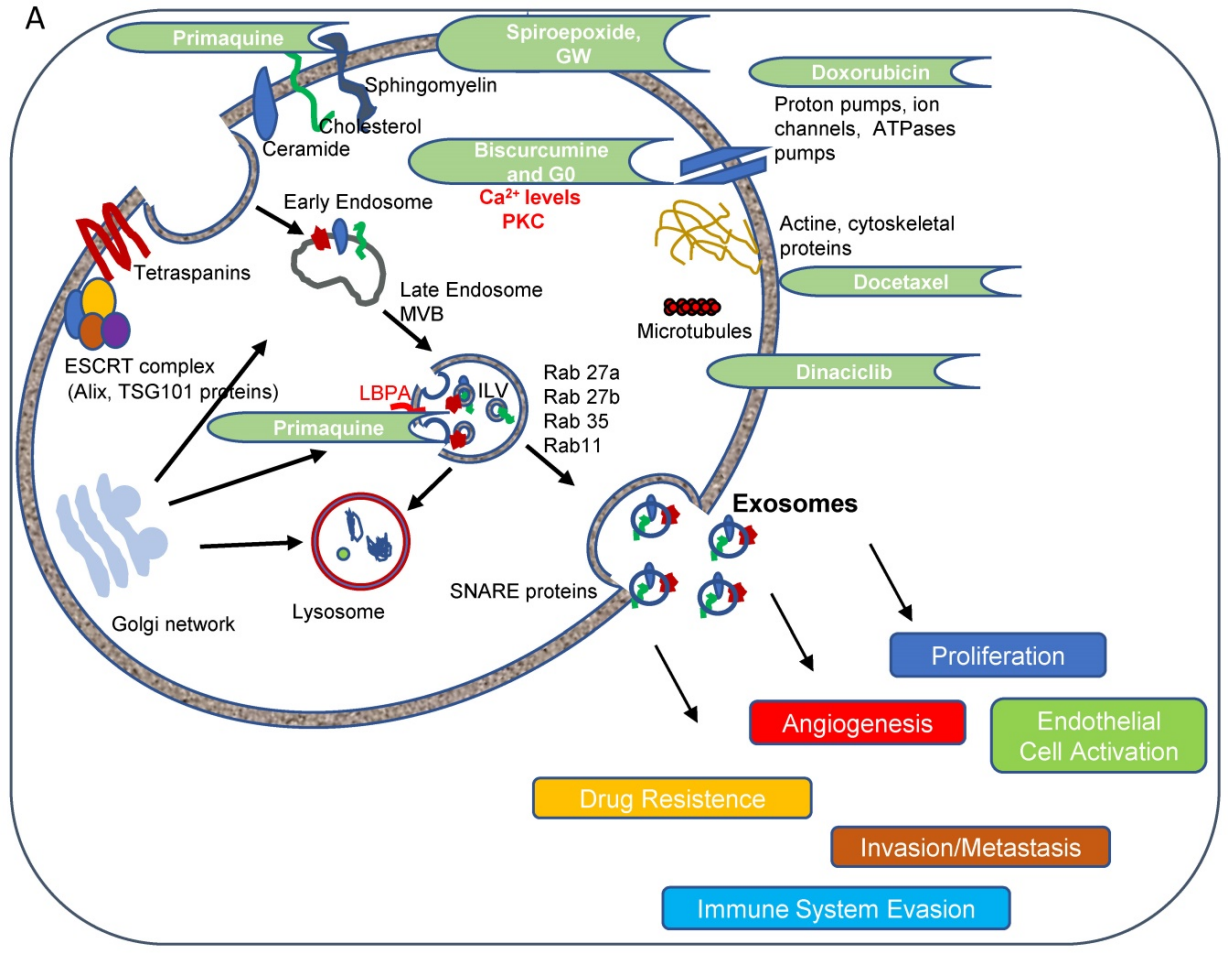

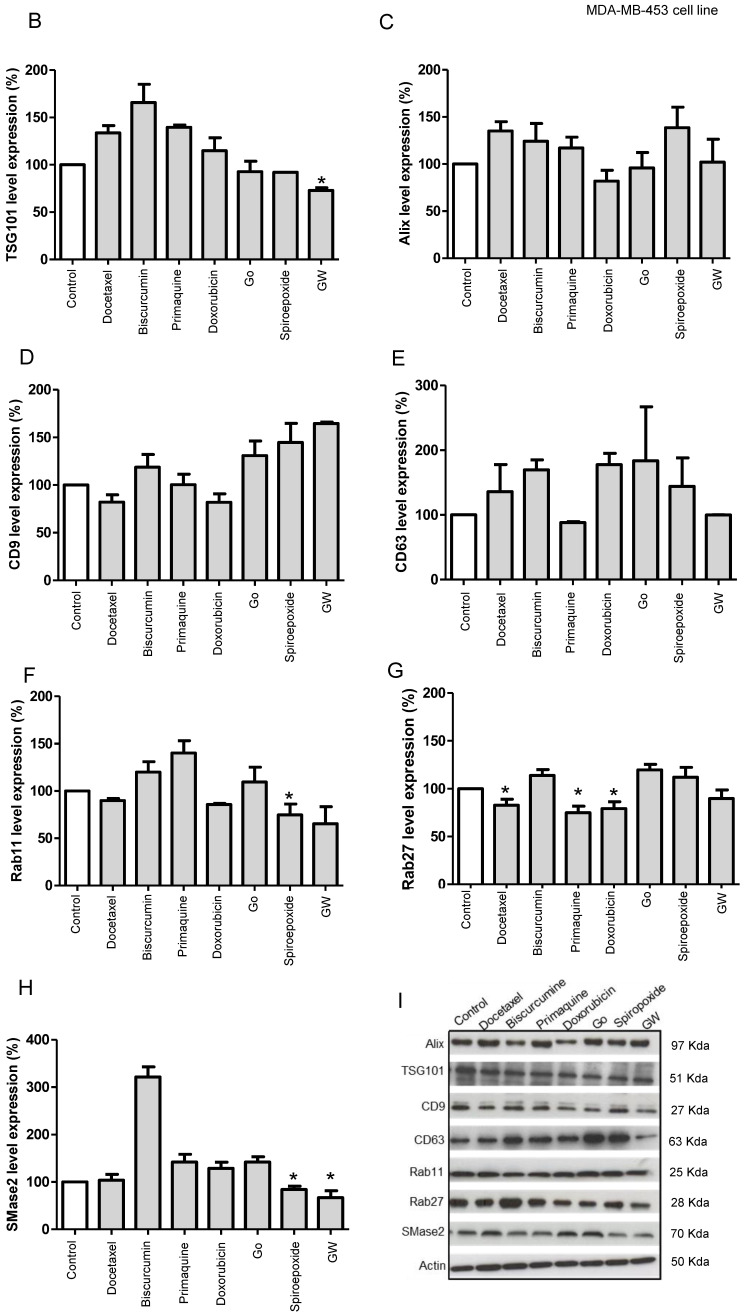

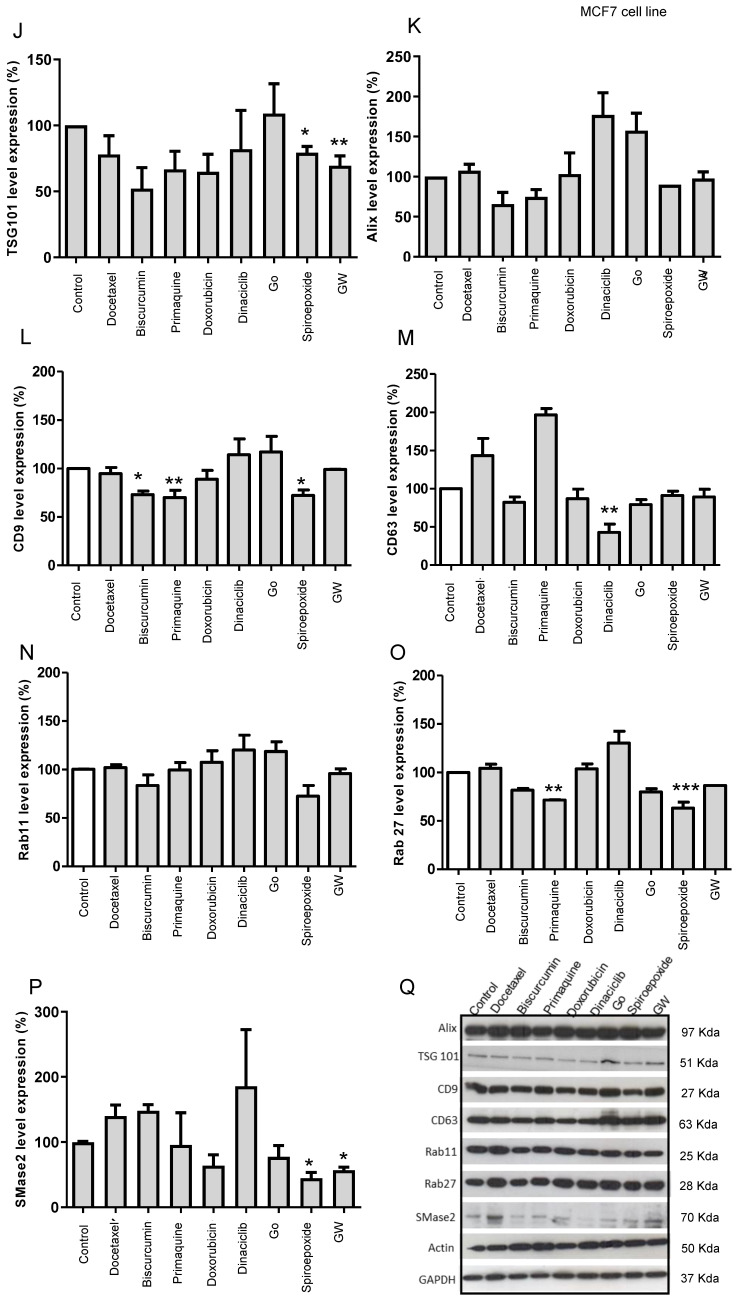
** (A) Targets of Inhibitors of Exosome Biogenesis and Release.** The ESCRT complex is one of the most studied pathways involved in exosome biogenesis; however, tetraspanins, sphingomyelinases, and lipids (such as ceramide and LBPA) take part in the membrane invagination of ILV and represent ESCRT-independent pathways in exosome biogenesis. The mechanisms that regulate exosome release via MVB fusion with the plasma membrane remain incompletely understood, although intracellular calcium changes, cell depolarization induced by potassium ions, and microtubule/cytoskeletal rearrangements have been reported to be involved in exosome release. Small GTPases of the Rab family and SNARE complexes are often involved in the intracellular trafficking and fusion of compartments and play a role in exosome release. Tumor-secreted exosomes promote proliferation, angiogenesis, invasion, and metastasis, among other effects in target cells. The figure depicts how docetaxel, doxorubicin, biscurcumin, dinaciclib, and primaquine could interfere with exosome biogenesis/release.** (B-Q) Modulation of ESCRT-dependent and -independent Exosome Biogenesis/Release Pathways by Identified Small Molecule Inhibitors in Breast Cancer Cells.** Representative histograms depicting protein analysis of **(B-H)** MDA-MB-453 (n=3) and **(J-P)** MCF7 (n=5) cell extracts following treatment with small molecule inhibitors. Representative Western blots for **(I)** MDA-MB-453 and **(Q)** MCF7 cell extracts. Data shown as mean ± SEM. Statistics employed ANOVA Dunnett's Multiple Comparison test relative to control (DMSO). *p<0.05, **p<0.01, ***p<0.001.

We finally attempted to understand the underlying molecular mechanisms involved in exosome inhibition with future therapeutic or diagnostic applications in mind. We analyzed TSG101 and Alix (ESCRT dependent pathway) and CD9, CD63, and N-SMase2 (ESCRT independent pathway), protein levels post-treatment as essential factors in exosome biogenesis pathways, and Rab11 and Rab27 as critical factors in vesicular trafficking and release pathways **(Figure 5)**. **Table S3** summarizes the therapeutic relevance of the small molecule hits identified with regard to their application in advanced breast cancer and the potential molecular targets with regard to exosome inhibitors (**Figure 5A**).

Our Western blotting studies in MDA-MB-453 cells (**Figure 5B-I,** representative Western blot in **I**) revealed that GW4869 and spiroepoxide (positive controls) negatively regulated SMase2 levels significantly, in agreement with their roles as inhibitors of this enzyme (**Figure 5H)** 42. These inhibitors also modulated TSG101 levels, although only GW4869 induced a significant change, suggesting that the inhibition of exosome biogenesis could function via an SMase2 or ESCRT-dependent pathway. Docetaxel and doxorubicin treatment also induced the modulation of CD9 tetraspanin expression and significantly downregulated the expression of Rab27. We also discovered that primaquine treatment significantly downregulated Rab27 and modulated the levels of CD63. Overall, this data agrees well with our prediction from low/low ExoScreen/LBPA signals; furthermore, we noted that GW4869, spiroepoxide, docetaxel, and doxorubicin also impacted Rab11 expression in MDA-MB-453 cells.

Data obtained from MCF7 cells (**Figure 5J - Q**, representative Western blot in **Q**) for GW4869 and spiroepoxide showed similar behavior regarding SMase2 as a target **(Figure 5P**); however, biscurcumin and primaquine functioned in a cell-specific manner to significantly modulate TSG101 levels **(Figure 5J)**. Furthermore, spiroepoxide biscurcumin and primaquine significantly modulated CD9 tetraspanin levels **(Figure 5L)**, while biscurcumin, primaquine, and doxorubicin modulated ALIX levels **(Figure 5K),** although this failed to reach significance. Dinaciclib treatment also significantly downregulated CD63 tetraspanin levels **(Figure 5M)**, while spiroepoxide and primaquine significantly diminished the expression of Rab27 levels **(Figure 5O)**.

Overall, we provide evidence that exosome inhibitors employ different molecular mechanisms in a cell-specific manner, with MCF7 cells appearing to display a higher sensitivity to exosome inhibitors than MD-MBA-453 cells; however, we note the need for further studies to fully explore and understand the molecular mechanisms at play.

## Discussion

Exosomes, intercellular communicators released in huge numbers by cancer cells 2, display enrichment in specific tetraspanins (e.g., CD9 and CD63) and the unconventional phospholipid LBPA when compared to other EV populations 1, 24, 27, 28, 36. During cancer progression, exosomes promote the formation of pre-metastatic niches and induce drug resistance through several mechanisms 6, 9, 10, 47-51; therefore, inhibiting tumor-derived exosome biogenesis/release or even uptake within pre-metastatic sites could represent a crucial part of the design of personalized combination therapies that prevent cancer progression or recurrence. The inhibition of HER2-positive breast cancer progression by decreasing the biogenesis/release of exosomes has already been reported 50**;** however, many obstacles to research and clinical translation remain in place.

As shown in** Figure 5A,** exosome biogenesis/release involves a multitude of factors whose exact roles remain unclear 41. Further exploration of said roles may aid the identification of small molecule inhibitors that modulate exosome biogenesis/release and contribute to the development of personalized combination therapies for cancer treatment; however, the current methodologies employed to purify and quantify exosomes are time-consuming and challenging to translate into clinical practice 1. Moreover, the most common protocols to isolate exosomes also co-purify vesicles from endosomal and other origins; consequently, different isolation methods influence the nature of EV populations 52. The absence of specific exosome markers represents another critical problem related to exosome purification. Many proteins used as exosome markers (such as MHC class I and II molecules, heat shock proteins, and flotillins) are also present in other EV populations and cannot be considered exosome specific 20; therefore, the identification and quantification of exosomes overall in clinical samples remain challenging.

This study describes an HTS-compatible convergent approach (by combining ExoScreen and InCell® Analyzer) to accelerate the identification of small molecules that modulate tumor-derived exosome levels. ExoScreen is a sensitive and rapid analytical technique for profiling circulating EVs directly from blood samples that has been validated in patients with colorectal cancer 34. We have taken advantage of ExoScreen to search for small molecule inhibitors of exosomes in breast cancer cells and combined ExoScreen with LBPA immunodetection to provide additional information on the intracellular mechanisms of exosome biogenesis. This combined approach can help to discover therapeutic approaches to modulate the levels of tumor-derived exosomes and identify their primary molecular mechanisms of action - exosome release or biogenesis - in the same assay (**Figure 1**). Importantly, we can achieve this information without needing validation experiments, which are essential in strategies using GFP-CD63 as a marker 53. In this case, the authors required an extracellular vesicle purification step that used ultracentrifugation followed by subsequent quantification by qNano-IZON particle quantitative analysis to better understand what occurs in the extracellular space to avoid any possible data misinterpretation 53. These additional assays require an investment of time and extra resources; alternatively, our convergent approach provides extracellular (ExoScreen 34) and intracellular (in Cell® LBPA quantification 23) information simultaneously and establishes whether the small molecules under consideration influence exosome release or biogenesis mechanisms. This strategy provides an improved perspective on how drugs function, which can contribute to the design of efficient combination therapies; however, we note that further studies will be necessary to deepen our understanding of the underlying molecular mechanisms.

We optimized the already reported ExoScreen assay 34 to detect a CD9/CD63-expressing population of exosomes (extracellular event), thereby bypassing time-consuming purification steps in comparison with the classical approaches (UC isolation and NTA characterization). CD9 is commonly present in exosomal populations (although may be present in other EVs), while CD63 enrichment occurs in late endosomes 54 and has been employed to detect endosome-derived exosomes 20. Therefore, we believe that the combination of CD9 and CD63 tetraspanins represents a robust means to isolate exosomes from other vesicular populations. Of note, cancer-related studies have previously exploited the presence of CD63 on exosomes to perform HTS to identify inhibitors of exosome biogenesis/release 53. We note that the antibodies used for the ExoScreen evaluations allow the visualization of the CD9/CD63 expressing population of exosomes; however, we can design the assay to discriminate other populations of EVs, including CD9/CD81- or CD63/CD81-expressing populations, corresponding to larger EVs, or integrate specific biomarkers of other diseases, offering a much broader application spectrum to this convergent strategy.

In parallel, we also monitored endosomal-restricted LBPA expression (intracellular event). Endosomal-restricted LBPA expression supports the formation of the ILVs that become released into the extracellular space as exosomes. The limitations associated with LBPA immunostaining include the association of LBPA with a small pool of lysosome-targeted ILVs and the involvement of LBPA in cholesterol biogenesis; however, the high levels of LBPA in exosomes could help to differentiate ILVs/exosomes from other EVs that bud from the plasma membrane 23. Given this knowledge, we optimized the detection of LBPA by immunofluorescence using InCell® technology to further explore the modulation of exosome biology by selected clinically used FDA-approved drugs. All selected small molecules presented molecular mechanism of action known to alter exosome release/biogenesis pathways 41; therefore, our approach could lead to drug repurposing, accelerating the progress of identified exosome inhibitors towards their clinical use as anti-cancer/antimetastatic agents. Of note, we worked at concentrations that ensured the absence of toxicity in selected breast cancer cells. We highlight the complementary nature of detecting CD9 and CD63 by ExoScreen technology and LBPA by InCell® technology and not any direct proportionality in quantitative terms. TEM images supported the intracellular and extracellular events analyzed by ExoScreen and the LBPA immunostaining following treatments.

Following the optimization of both independent technologies with well-identified exosome modulating drugs, results revealed that docetaxel, biscurcumin, primaquine, and doxorubicin functioned as exosome inhibitors in Her-2 positive MDA-MB-453 cells and hormone-dependent luminal A MCF7 cells and dinaciclib only in MCF7 cells. While these FDA-approved small molecules are commonly used in the clinic, some even as cancer treatments, we now suggest their role as exosome inhibitors that may potentially impact metastatic progression. As the mechanisms responsible for biogenesis/release influence the cargo packaged into exosomes, our approach may also contribute toward the characterization of exosome contents, which carries prognostic and diagnostic clinical value 52, 55, 56. In comparison with the more commonly employed isolation (UC) and characterization (NTA) approaches, ExoScreen selected a defined exosomal population, which provides a more accurate analysis of the function of potential inhibitors compared to the heterogeneous EV/exosome populations isolated using classical approaches 20, 57. Furthermore, incorporating LBPA signal detection affords additional information.

In conclusion, we identified FDA-approved small molecules commonly used in the clinics as exosome inhibitors in MDA-MB-453 and MCF7 breast cancer cells through an ExoScreen-based methodology, where the changing levels of exosomes and the mechanisms of action involved can be monitored using InCell^®^ analysis. Our strategy could allow drug repurposing in preventive/treatment strategies for primary breast cancers and associated metastasis; therefore, our findings may improve diagnostic and therapeutic approaches to cancer and other diseases/disorders. Furthermore, we propose LBPA as an efficient means to characterize the exosome population combined with other more widely used markers (e.g., tetraspanins). In a study by Rabia et al. 33, the authors described the utility of LBPA as a biomarker of urinary exosomes to identify endo-lysosomal dysfunction. The combination of LBPA with other proteins enriched in exosomes such as tetraspanins (e.g., CD9 or CD63) could be used in the design of a biomarker panel for a liquid biopsy that would allow disease monitoring with regards to treatment response and the prediction of possible relapses/recurrence or metastatic progression. We anticipate that our findings may pave the way for the development of HTS approaches, applications in clinical settings, and the design of novel therapeutic approaches capable of modulating tumor microenvironment and enhancing the armory of pharmacological anti-cancer strategies.

## Supplementary Material

Supplementary figures and tables.

## Abbreviations

BMP: phospholipid bis(monoacylglycero)phosphate
BSA: bovine serum albumin
DMEM/F12: Dulbecco's Modified Eagle's Medium F12
DMSO: dimethyl sulfoxide
emi: emission
EV: extracellular vesicle
ER: estrogen receptor
exc: excitation
ESCRT: endosomal sorting complexes required for transport
FBS: fetal bovine serum
FITC: fluorescein isothiocyanate
Go: Go6983
GW: GW4869
HER2: erbB-2 receptor tyrosine protein kinase
HRP: Horseradish peroxidase
HTS: high through screening
ILV: intraluminal vesicle
LBPA: lysobisphosphatidic acid
MHC class II and I: major histocompatibility complex molecules I and II
MVB: multivesicular body
nSMase: neutral sphingomyelinase
NTA: nanoparticle tracking analysis
PB: phosphate buffer
PBS: phosphate buffer saline
PDCD6IP: programmed cell death 6 interacting protein
PKC: Protein kinase C
PR: progesterone receptor
RT: room temperature
SDS PAGE: sodium dodecyl sulfate polyacrylamide gel electrophoresis
TME: transmission electron microscopy
TRITC: tetramethylrhodamine
TSG101: tumor susceptibility gene 101
UC: ultracentrifugation
