# Supplementary material for: A Rapid, Convergent Approach to the Identification of Exosome Inhibitors in Breast Cancer Models
Source: Nanotheranostics. 2023 Jan 1;7(1):1–21. doi: 10.7150/ntno.73606 (PMC9760366; doi:10.7150/ntno.73606)
Supplement: Supplementary file 1 — Supplementary figures and tables. [file ntnov07p0001s1.pdf]

Supporting Information

A Rapid, Convergent Approach to the Identification of Exosome Inhibitors in Breast Cancer Models

Zoraida Andreu*, Esther Masiá, David Charbonnier, and María J. Vicent*

Polymer Therapeutics Lab., Prince Felipe Research Center (CIPF), C. Eduardo Primo Yúfera 3, 46012, Valencia, Spain

* Correspondence to be addressed to

zandreu@fivo.org and mjvicent@cipf.es.

Phone: +34963289680 (Ext2307#)

Supplementary Materials and Methods

Cell Culture and ExoScreen Assay Optimization

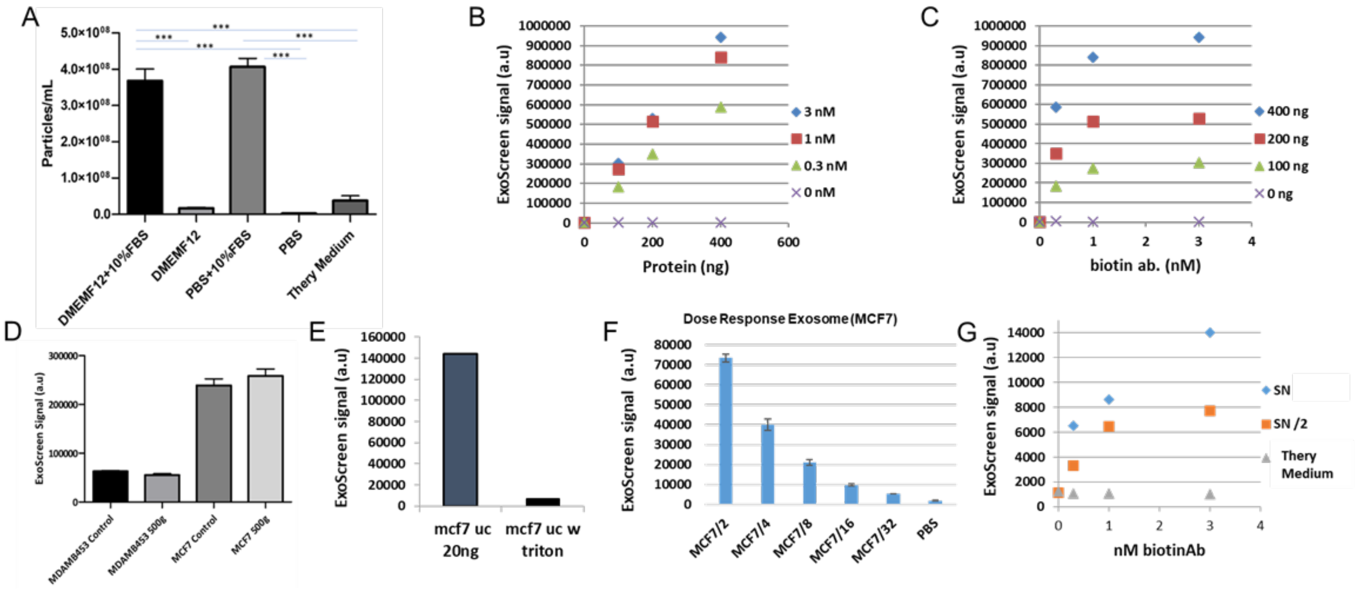

Figure S1. Cell culture and ExoScreen assay optimization. (A) Control particle number measurement using nanoparticle tracking analysis (NTA) in different cell media. The protocol implemented for depleting exosomes from culture medium followed the well-accepted protocol published by Thery et al. [1]. Initially, the culture medium containing 20% FBS was centrifuged for 17 hours at 100,000 × *g*, 4°C, and the supernatant (i.e., depleted medium) was filter sterilized. Then, the growth medium was completed by adding fresh medium to reduce 20% FBS to 10% FBS. Before starting any experiment, several types of serum and medium were evaluated to select the optimal choice. The results of these comparisons provide robust evidence that the medium used in our assays ("Thery medium") is adequate for purpose. **(B and C)** ExoScreen signal from pure exosome protein dose-response curve (MCF7 cell line) measured with different amounts of biotinylated CD63-antibody. (**C**) Shows the optimal linear working range between [0.3-1 nM]. **(D)** ExoScreen analysis of control samples in MCF7 and MDA-MB-453 breast cancer cell lines (n=6 culture wells, duplicates intra-assay). Before (control) and after centrifugation (500g, 10 min). NO differences where observed indicating the absence of debris interference in the readout **(E)** ExoScreen signal from 20 ng pure exosome protein of MCF7 cells in the presence or absence of triton. The loss of exosome membrane integrity (by adding triton) promotes a drastic decrease in the ExoScreen signal, indicating that small factors in the supernatant do not interfere with the signal. **(F)** ExoScreen signal from serial dilutions (1/2) of pure exosomes ($7.77*10^9$ exosomes/mL; NTA) from MCF7 cells using 0.3 nM biotinylated CD63-antibody. **(G)** ExoScreen signal from serial dilutions (1/2) of supernatant (SN) from MCF7 cell line cell culture by using different amounts of biotinylated CD63-antibody.

LBPA Immunostaining Using InCell® Analyzer

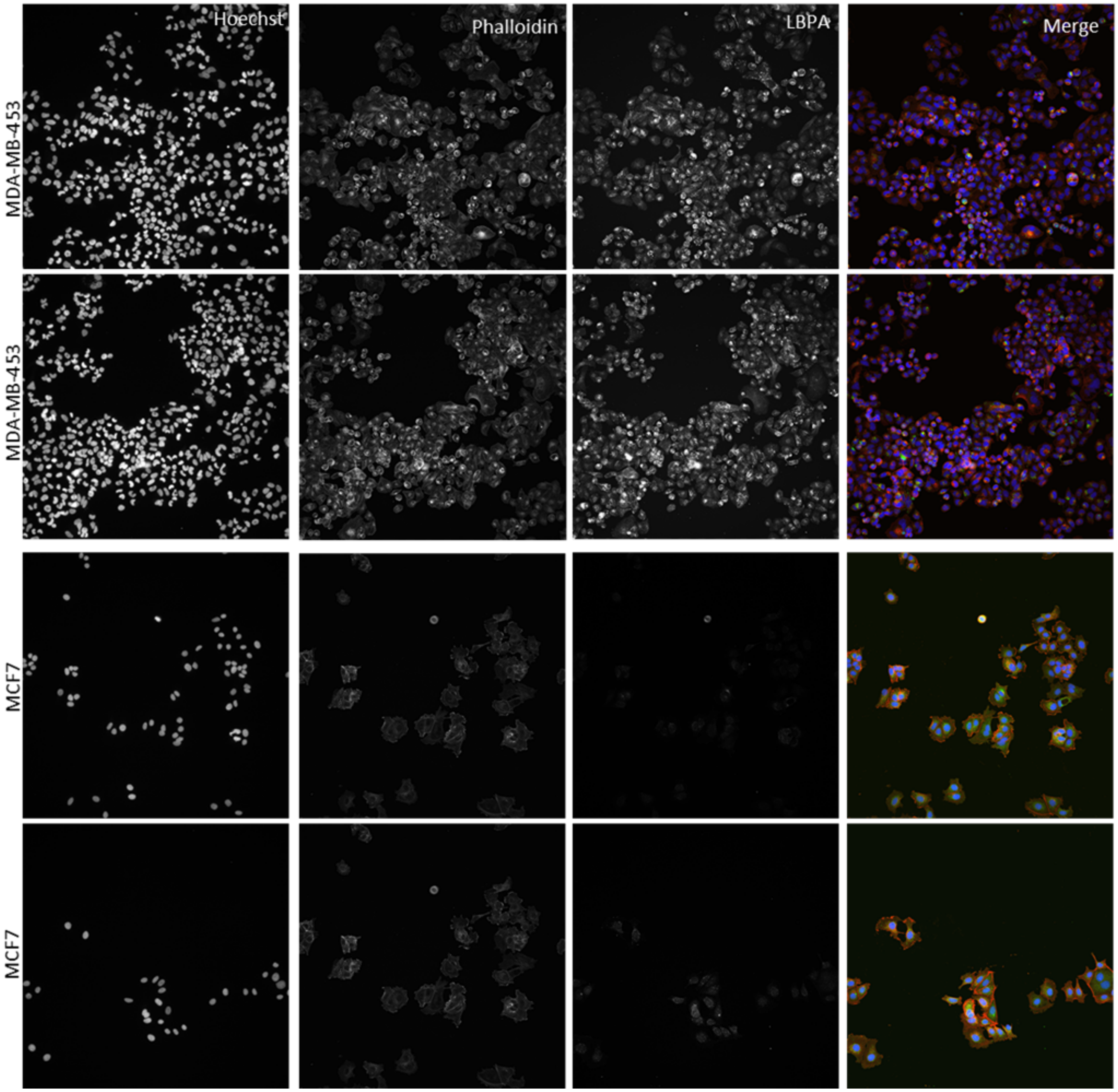

Figure S2. Representative Images of Workflow Segmentation in LBPA assay. Hoechst and phalloidin were used to segment the nuclei and the cytoplasm, respectively, to support the acquisition of images in the InCell® Analyzer 2200 and analyze images in the Developer Toolbox software. The LBPA granules were segmented using the FITC signal, and these granules were associated with the cell segmentation to quantify the granules inside the cells and avoid the appearance of artifacts in the analysis. Scale bar = 100 μm.

Supplementary Results

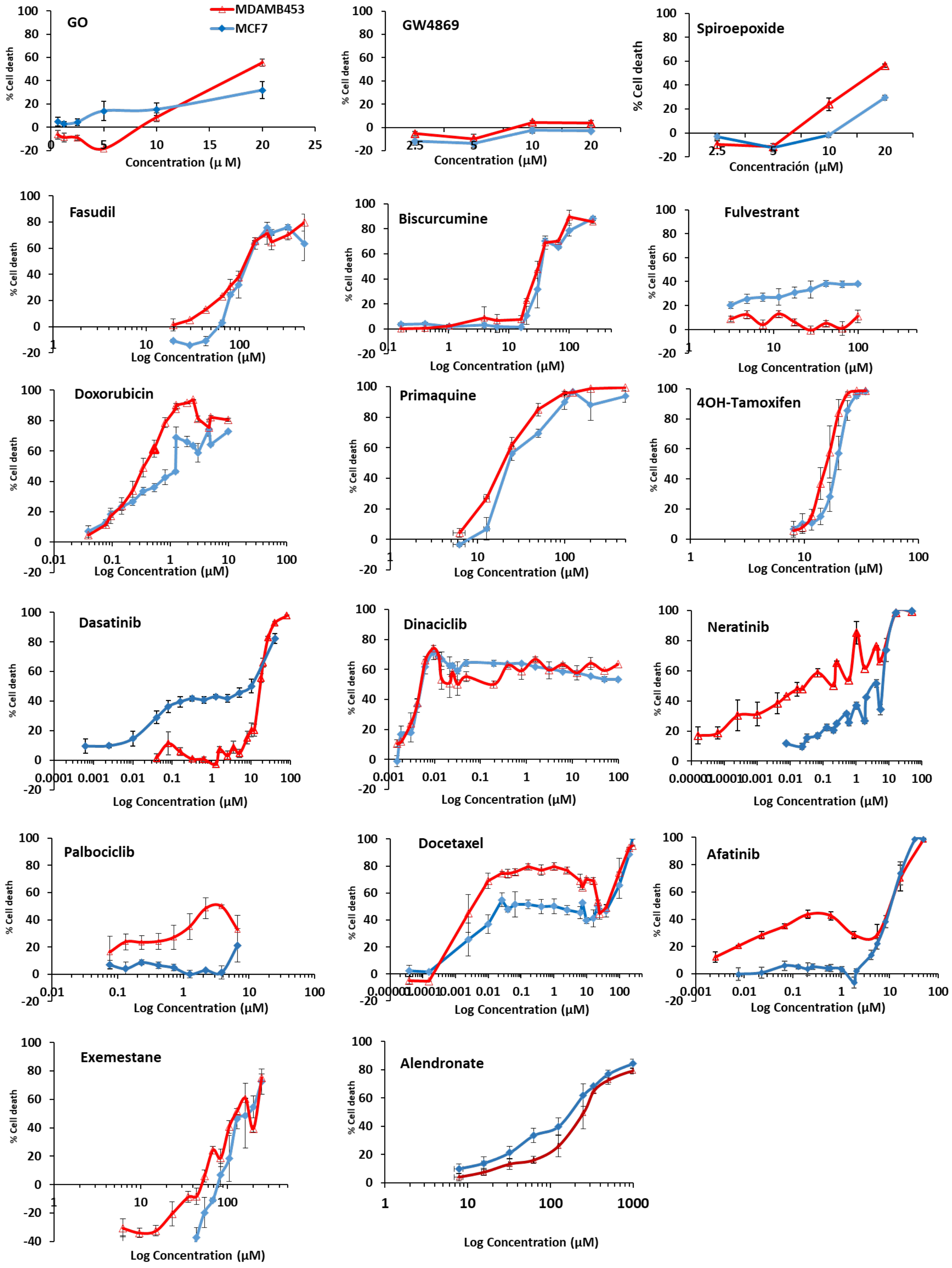

Figure S3. Cell Viability Study for Selected FDA-approved drugs and Control Compounds. Cell viability measured by CellTiter 96® Aqueous Non-Radioactive Cell Proliferation Assay (72h post-incubation) against HER2-positive MDA-MB-453 (red) and luminal A MCF7 (blue) breast cancer cell lines. Each point is representative of the mean ± SEM of triplicate samples.

Table S1. Detailed information on the screened compounds in the HER2-positive MDA-MB-453 breast cancer cell line. The concentration was based on cell viability data (**Figure S3**), and results were obtained following our convergent approach (ExoScreen + LBPA immunostaining by InCell®).

Compound Name	Concentration (μM)	Cell Toxicity (% control)	ExoScreen Signal	LBPA Signal	Mode of Action regarding Exosome Modulation[$]
**G0 (Control)**	5	0	Low	High	Inhibitor of exosome release
**Spiroxide (Control)**	6	0	Low	Low	Inhibitor of exosome biogenesis
**Docetaxel**	$0.2 \cdot 10^{-3}$	<17	Low	High	Inhibitor of exosome release
**Biscurcumin**	6	7	Low	High	Inhibitor of exosome release
**Primaquine**	6.25	4	Low	High	Inhibitor of exosome release
**Doxorubicin**	0.02	<5	Low	High	Inhibitor of exosome release
**Dinaciclib**	$1 \cdot 10^{-3}$	<11	High	High	Activator of exosome biogenesis
**Exemestane**	10	0	High	Low	Activator of exosome release
**Palbociclib**	0.02	<20	High	Low	Activator of exosome release
**Alendronate**	90	<20	High	High	Activator of exosome biogenesis
**Fasudil**	20	1	High	High	Activator of exosome biogenesis
**Dasatinib**	5	5	High	High	Activator of exosome biogenesis
**Neratinib**	$0.016 \cdot 10^{-3}$	<17	High	High	Activator of exosome biogenesis
**Tamoxifen**	7	0	High	High	Activator of exosome biogenesis
**Afatinib**	$1.5 \cdot 10^{-3}$	<12	High	NC	Activator of exosome biogenesis and/or release
**Fulvestrant**	23	0	High	NC	Activator of exosome biogenesis and/or release

[$]Results obtained from the convergent approach implementing our Combinatorial Signal Approach criteria (**Figure 1**). NC=no change observed relative to control (considered as controls cells without treatment)

Table S2. Detailed information on the screened compounds in the HER2-positive MDA-MB-453 breast cancer cell line. The concentration was based on cell viability data (**Figure S3**), and results were obtained following our convergent approach (ExoScreen + LBPA immunostaining by InCell®).

**Compound Name**	**Concentration** (µM)	**Cell Toxicity (% control)**	**ExoScreen Signal**	**LBPA Signal**	**Mode of Action regarding Exosome Modulation**[$]
**G0 (Control)**	2.5	5	Low	High	Inhibitor of exosome release
**Spiroxide (Control)**	6	0	Low	Low	Inhibitor of exosome biogenesis
**Docetaxel**	$0.2 \cdot 10^{-3}$	<17	Low	High	Inhibitor of exosome release
**Biscurcumin**	6	2	Low	High	Inhibitor of exosome release
**Primaquine**	6.25	0	Low	High	Inhibitor of exosome release
**Doxorubicin**	0.02	<7	Low	High	Inhibitor of exosome release
**Dinaciclib**	$1 \cdot 10^{-3}$	0	Low	High	Inhibitor of exosome release
**Exemestane**	7	0	High	Low	Activator of exosome release
**Palbociclib**	0.01	<15	High	Low	Activator of exosome release
**Alendronate**	10	<14	High	High	Activator of exosome biogenesis
**Fasudil**	30	0	High	High	Activator of exosome biogenesis
**Dasatinib**	$0.6 \cdot 10^{-3}$	<10	High	High	Activator of exosome biogenesis
**Neratinib**	0.02	<12	High	High	Activator of exosome biogenesis
**Tamoxifen**	1	0	High	High	Activator of exosome biogenesis
**Afatinib**	3	<14	High	Low	Activator of exosome release
**Fulvestrant**	3	<20	High	Low	Activator of exosome release

[$]Results obtained from the convergent approach implementing our Combinatorial Signal Approach criteria (**Figure 1**). NC=no change observed relative to control (considered as controls cells without treatment)

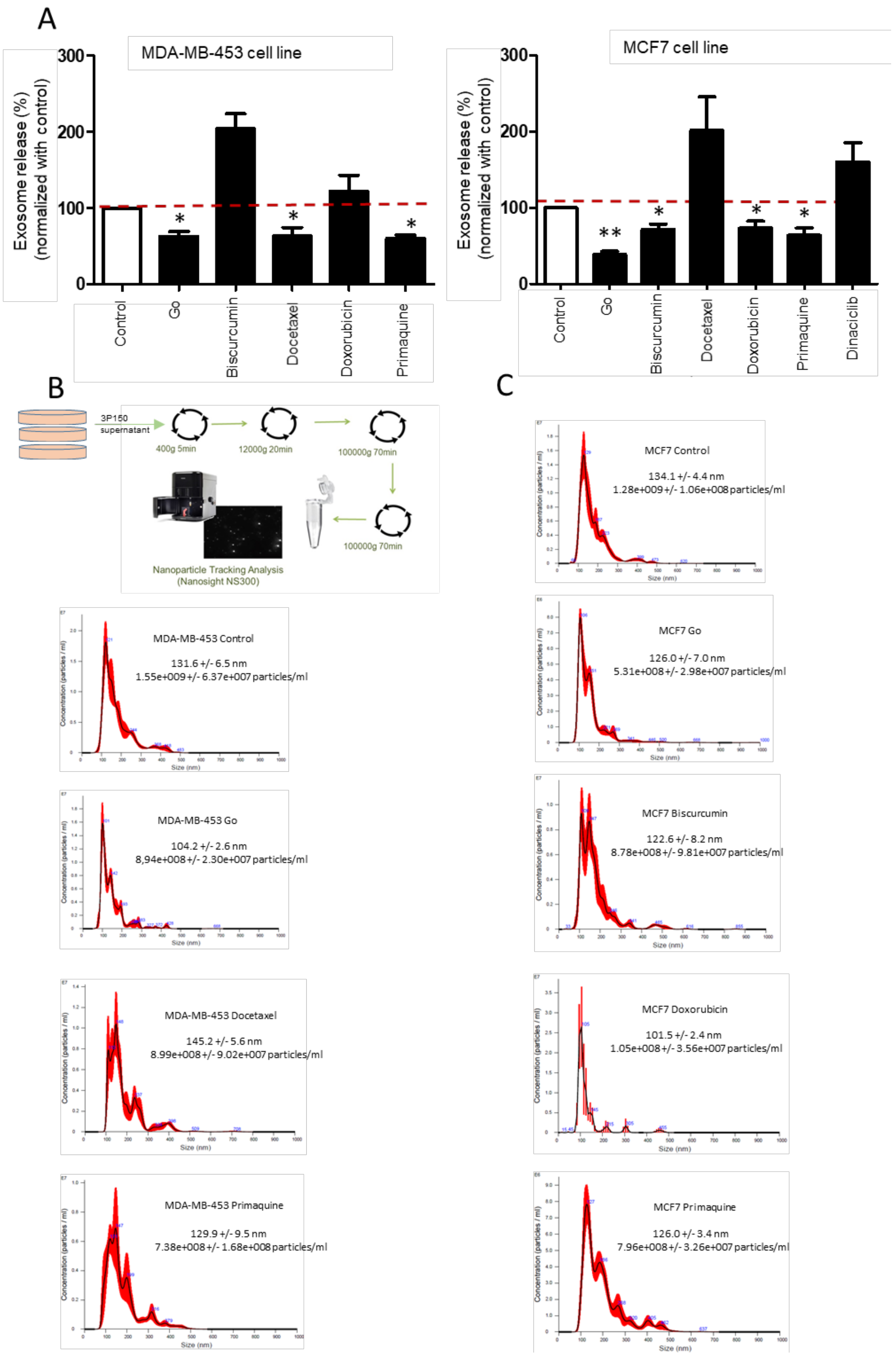

A
MDA-MB-453 cell line
Exosome release (%) (normalized with control)
300
200
100
0
Control
Go
Biscurcumin
Docetaxel
Doxorubicin
Primaquine
MCF7 cell line
Exosome release (%) (normalized with control)
Dinaciclib
B
3P150 supernatant
400g 5min
12000g 20min
100000g 70min
100000g 70min
Nanoparticle Tracking Analysis (Nanosight NS300)
C
MCF7 Control
134.1 +/- 4.4 nm
1.28e+009 +/- 1.06e+008 particles/ml
MCF7 Go
126.0 +/- 7.0 nm
5.31e+008 +/- 2.98e+007 particles/ml
MCF7 Biscurcumin
122.6 +/- 8.2 nm
8.78e+008 +/- 9.81e+007 particles/ml
MCF7 Doxorubicin
101.5 +/- 2.4 nm
1.05e+008 +/- 3.56e+007 particles/ml
MCF7 Primaquine
126.0 +/- 3.4 nm
7.96e+008 +/- 3.26e+007 particles/ml
MDA-MB-453 Control
131.6 +/- 6.5 nm
1.55e+009 +/- 6.37e+007 particles/ml
MDA-MB-453 Go
104.2 +/- 2.6 nm
8,94e+008 +/- 2.30e+007 particles/ml
MDA-MB-453 Docetaxel
145.2 +/- 5.6 nm
8.99e+008 +/- 9.02e+007 particles/ml
MDA-MB-453 Primaquine
129.9 +/- 9.5 nm
7.38e+008 +/- 1.68e+008 particles/ml
Concentration (particles / ml)
Size (nm)

Figure S4. Comparison of Inhibitor Candidates by Nanoparticle Tracking Analysis (NTA) after Purification by UC. MDA-MB-453 and MCF7 cell lines were grown in exosome-free media and treated with drugs for 72 hours. (**A**) Exosomes isolated by UC and concentration as measured by NTA. These conventional techniques confirm that docetaxel and primaquine in MDA-MB-453 cells and biscurcumin, doxorubicin, and primaquine in MCF7 cells function as exosome release inhibitors. Data shown as the percentage of exosome release normalized to the control sample (100%), mean ± SEM of three independent experiments. Exosome number normalized by cell number in each condition. ANOVA, Dunnett's comparison Multiple Comparison test relative to control condition * $p<0.05$, ** $p<0.01$. Go6983 was used as an inhibitor reference control. **(B)** Workflow. (**C**) Representative plots of NTA measurements. Mean particle size ± SD and mean concentration ± SD are shown from five videos recorded for each exosome sample.

Table S3. Additional Information on the identified Exosome Inhibitors in Breast Cancer Cells

Compound Name (PubChem)	Structure	Therapeutic Information	BC Cell line target	Target as exosome inhibitor
Biscurcumin $C_{21}H_{20}O_6$	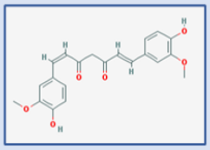	Inhibit phorbol ester-induced protein kinase C (PKC) activity	MCF7 (Luminal A, ER+PR+HER-) 453 (ER-PR-HER+)	PKC
Docetaxel $C_{43}H_{53}NO_{14}$	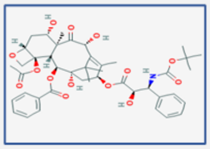	Antineoplastic agent. Inhibitor of cellular mitosis and that currently plays a central role in the therapy of many solid tumor including breast and lung cancer. Docetaxel binds specifically to the beta-tubulin subunit of microtubules resulting in the persistence of aberrant microtubule structures.	MCF7 (Luminal A, ER+PR+HER-) 453 (ER-PR-HER+)	Cytoskeletal protein network
Doxorubicin $C_{27}H_{29}NO_{11}$	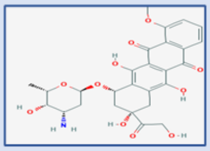	Interfere in calcium regulations and the Na+/K+ pumps. Promotes the inhibition of Na+/H+ and Na+/ Ca2+ channels or of H+ pump	MCF7 (Luminal A, ER+PR+HER-) 453 (ER-PR-HER+)	Proton pumps/Ca2+ regulation
Dinaciclib $C_{21}H_{28}N_6O_2$	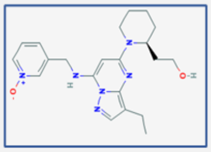	Antineoplastic activity. Inhibits cyclin dependent kinases CDK1, CDK2, CDK5, and CDK9; inhibition of CDK1 and CDK2 may result in cell cycle repression and tumor cell apoptosis.	MCF7 (Luminal A, ER+PR+HER-)	Microtubule network
Primaquine $C_{15}H_{21}N_3O$	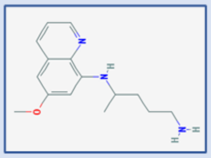	A potent therapeutic agent (malaria treatment). Its mechanism of action still lacks a more detailed understanding at a molecular level. Molecular mechanism of primaquine-lipid interaction has been described so this drug has an effect on the dynamic structure of lipid model membranes.	MCF7 (Luminal A, ER+PR+HER-) 453 (ER-PR-HER+)	Lipid rafts

Role of Tumor-derived Exosomes in Cancer Cell Migration. Migration Assays

To highlight the importance of tumor-derived exosome inhibition to slow/stop cancer progression and metastasis, we performed a simple migration assay using human foreskin fibroblasts. *In vitro* "scratch" experiments using human foreskin primary fibroblasts, kindly donated by Dr. Pilar Sepúlveda (La Fe Hospital, Valencia, Spain), were performed to analyze the role of exosomes in cell migration as described in Liang et al. with some minor modifications [2]. Briefly, fibroblasts were seeded in 12-well plates at a concentration of 75,000 cells/well in basal medium (DMEM F12 High Glucose plus 10% exosome-free FBS, Sigma Life Sciences, Germany). Once confluent (24 h after culture), a straight scratch was made through the fibroblast monolayer with a 10 µl pipette tip. Cells were then washed with 1 ml of growth medium to remove cell debris and smooth the edge of the scratch and then replaced with 1 ml of DMEM F12 High Glucose plus 10% exosome-free FBS. 5 µg of exosomes purified by differential ultracentrifugation (UC) from MDA-MB-453 and MCF7 cell lines were added (n=3). The capacity of the fibroblasts to migrate and invade the scratch was tracked using time-lapse microscopy (Leica DMI6000 automatic inverted microscope with Leica Application Suite, Advance Fluorescence Lite 2.6.0 build 7276 AF 6000 MS for life cell time-lapse experiment), which acquired images every 20 min for 24 h from two different fields in each well. The scratch width in µm was measured from images taken from 0 h to 15 h (as a representative final migration time) using the digital image processing program ImageJ 1.51j8 (National Institutes of Health, USA). Finally, the migration percentage was calculated as follows: (scratch width at t_0 – scratch width at 15 h) x 100 (**Figure S5A-B)**.

To verify the enhancement of fibroblast migration capacity by exosomes, Ki67 levels were evaluated by flow cytometry. After treatment, fibroblasts were collected by trypsin/EDTA solution (Thermo Fisher, USA), washed in PBS, centrifuged at 400g for 5 min at 4 ºC, and fixed in 4% PFA for 10 min for antibody immunostaining. For immunostaining, fibroblasts were washed in PBS and blocked in PBS+1% BSA+0.25% Triton for 1 h at RT. Cells were then incubated overnight with primary antibody mouse monoclonal anti-Ki67 1:75 (Millipore, Ref MAB 4190). Incubation with the secondary antibody Alexa 488 Goat anti-Mouse 1:500 (Abcam Ref A21121) was carried out for 1 h at RT. Data were then acquired using CytoFLEX S (Beckman Coulter) equipment and analyzed using CytExpert software (**Figure S5C**).

Fibroblasts incubated with exosomes displayed a higher migratory potential than untreated fibroblasts (**Figure S5A**-**B**). To discriminate the possible contribution of an exosome-mediated increase in fibroblast proliferation, we evaluated Ki67 staining in parallel by flow cytometry (**Figure S5C**). We failed to encounter any significant changes in fibroblast proliferation rate upon the addition of exosomes compared to untreated control fibroblasts, suggesting that exosome treatment impacted fibroblast migration independent of cell proliferation. Overall, these findings confirm the role of exosomes in cancer cell migration, suggesting the importance of identifying small molecule exosome inhibitors in cancer patients.

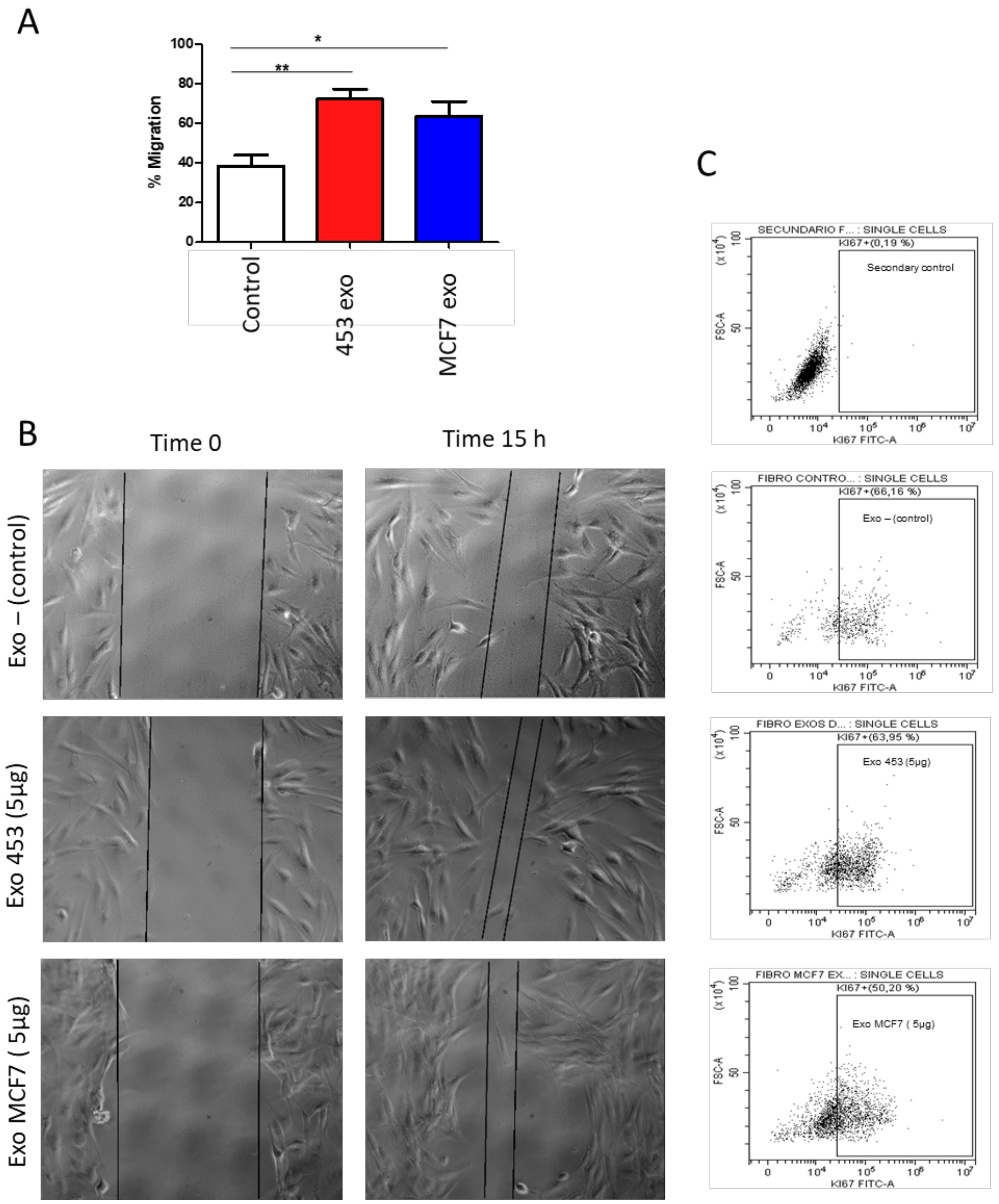

Figure S5. MDA-MB-453 and MCF7–derived exosomes increased fibroblast migration. (A) Percentage of fibroblast cell migration after treatment with 5 mg of exosome from breast cancer cells (n=3, also included control untreated wells). **(B)** Images from the scratch assay at 0 and 15 h. **(C)** Evaluation of Ki67 expression from exosome-treated fibroblasts by flow cytometry, CytoFLEX S (Beckman Coulter) equipment, and analyzed using CytExpert software. Data shown as mean ± SEM. Statistics employed ANOVA Dunnett's Multiple Comparison test relative to control (DMSO): *p<0.05, **p<0.01.

Cell Authentication

The human breast cancer cell lines MDA-MB-453 (ATCC HTB-131^{TM} - estrogen receptor (ER)-negative, progesterone receptor (PR)-negative, HER2-positive), and MCF7 (ATCC HTB-22^{TM} - ER+, PR+, HER-) were purchased from the American Type Culture Collection (ATCC, USA) and authenticated by cell genotyping at Eurofins Genomics, see the certificate below.

eurofins | Genomics

Eurofins Genomics Europe Applied Genomics GmbH, Anzinger Str. 7 a, D-85560 Ebersberg

Maria Helena Ferrandis
CENTRO DE INVEST PRINCIPE FELIPE
Carrer Eduardo Primo Yufera Cientific 3
46012 Valencia
Spain

Certificate
Cell Line Authentication Test
Order ID: 11107020120

Report date: 28.09.2020

Method:
DNA isolation was carried out from cell pellet (cell layer).
Genetic characteristics were determined by PCR-single-locus-technology.
16 independent PCR-systems D8S1179, D21S11, D7S820, CSF1PO, D3S1358, TH01, D13S317, D16S539, D2S1338, AMEL, D5S818, FGA, D19S433, vWA, TPOX and D18S51 were investigated.
(ASN-0002 core markers are colored grey, Thermo Fisher, AmpFISTR® Identifiler® Plus PCR Amplification Kit)
In parallel, positive and negative controls were carried out yielding correct results.

Result:

Client Sample Name	HCT-116	MDA-MB-231	MCF7	MCF10A	ZR-75-1	MDA-MB-453
Sample Code	CL00001640	CL00001646	CL00001647	CL00001648	CL00001649	CL00001650
D8S1179	12,14,15	13,13	10,14	14,16	11,13	10,12
D21S11	28,29,30	30,33.2	30,30	28,30	30,31,33.2	29,31
D7S820	11,12	8,8	8,9	10,11	8,10,11	10,10
CSF1PO	7,10,11	12,13	10,10	10,12	10,11,12,13	9,10,12
D3S1358	12,17,18,19	16,16	16,16	14,18	15,16	15,15
TH01	8,9	7,9.3	6,6	8,9.3	7,9.3	6,6
D13S317	10,12,13	13,13	11,11	8,9	9,13	12,12
D16S539	9,11,13	12,12	11,12	11,12	11,12	9,9
D2S1338	16,16	20,21	21,23	21,26	16,20,21,25	23,24
D19S433	11,12,13	11,14	13,14	13,15	11,13,14	13,14
vWA	17,18,22,23	15,15	14,15	15,17	15,16,18	17,18
TPOX	8,9	8,9	9,12	9,11	8,9	10,10
D18S51	15,16,17,18	11,16	14,14	18,19	11,13,14,16	15,20
AMEL	X,X	X,X	X,X	X,X	X,X	X,X
D5S818	10,11	12,12	11,12	10,13	12,13	11,11
FGA	18,22,23	22,23	23,24,25	22,24	20,22,23	18,23
Database Name	HCT 116*	89% identity: MDA-MB-231	MCF-7	MCF 10A	not in database	MDA-MB-453

The table shows the result of the cell line analysis and the comparison with the online database of the DSMZ (http://www.dsmz.de/de/service/services-human-and-animal-cell) and the Cellosaurus database (https://web.expasy.org/cellosaurus). Please note that only the PCR-systems according to ANSI/ATCC standard ASN-0002 were aligned (D5S818, D13S317, D7S820, D16S539, VWA, TH01, TPOX, CSF1PO, AMEL - colored grey).

*The sample could be identified as the stated cell line, although one or several PCR-Systems did show additional signals (see table). These signals could occur due to contamination of the cell line or possible mutations.

This report was created automatically and is therefore valid without a signature.

The laboratory is accredited acc. to **DIN EN ISO/IEC 17025:2005**. The accreditation applies only to the test methods specified in the accreditation certificate. All analyses have been carried out with greatest care and on the basis of state of the art scientific knowledge. The results refer solely to the analysed samples. The duplication and publication also in parts requires a written authorization by this laboratory. Our General Terms and Conditions apply exclusively and are available under eurofinsgenomics.com

Eurofins Genomics Europe Applied Genomics GmbH
Anzinger Straße 7 a
85560 Ebersberg
Germany

Tel.: +49 8092 8289-200
Fax: +49 8092 8289-201
Email: info-eu@eurofins.com
Web: eurofinsgenomics.com

Managing Directors: Dr. Michael Hadem,
Dr. Peter Persigehl
Register Court Munich HRB 207710
VAT ID: DE815473648

HypoVereinsbank
IBAN: DE23 2073 0017 7000 0006 50
SWIFT: HYVEDEMME17

References

1. Théry C, Amigorena S, Raposo G, Clayton A. Isolation and characterization of exosomes from cell culture supernatants and biological fluids. Current protocols in cell biology. 2006; Chapter 3: Unit 3.22.
2. Liang C-C, Park AY, Guan J-L. In vitro scratch assay: a convenient and inexpensive method for analysis of cell migration in vitro. Nature Protocols. 2007; 2: 329-33.
